# Use of antibiotics and asthma medication for acute lower respiratory tract infections in people with and without asthma: retrospective cohort study

**DOI:** 10.1186/s12931-019-1233-5

**Published:** 2020-01-06

**Authors:** Rachel Denholm, Esther T. van der Werf, Alastair D. Hay

**Affiliations:** 1 Centre for Academic Primary Care, Population Health Sciences, Bristol Medical School, University of Bristol, 39 Whatley Road, Bristol, BS8 2PS UK; 2 School of Medicine, Taylor’s University, Subang Jaya, Selangor Malaysia

**Keywords:** Asthma, Respiratory tract infections, Antibiotics

## Abstract

**Background:**

Antibiotics are overused in patients with acute lower respiratory tract infections (ALRTIs), but less is known about their use in patients with asthma, or the use of asthma medication for ALRTI in patients without asthma. Our aim was to describe the frequency, variation and drivers in antibiotic and asthma medication prescribing for ALRTI in adults with and without asthma in primary care.

**Methods:**

A retrospective cohort analysis of patients aged ≥12 years, diagnosed with an ALRTI in primary care in 2014–15 was conducted using data from the Clinical Practice Research Datalink. Current asthma status, asthma medication and oral antibiotic use within 3 days of ALRTI infection was determined. Treatment frequency was calculated by asthma status. Mixed-effect regression models were used to explore between-practice variation and treatment determinants.

**Results:**

There were 127,976 ALRTIs reported among 110,418 patients during the study period, of whom 17,952 (16%) had asthma. Respectively, 81 and 79% of patients with and without asthma received antibiotics, and 41 and 15% asthma medication. There were significant differences in between-practice prescribing for all treatments, with greatest differences seen for oral steroids (odds ratio (OR) 18; 95% CI 7–82 and OR = 94; 33–363, with and without asthma) and asthma medication only (OR 7; 4–18 and OR = 17; 10–33, with and without asthma).

Independent predictors of antibiotic prescribing among patients with asthma included fewer previous ALRTI presentations (≥2 vs. 0 previous ALRTI: OR = 0.25; 0.16–0.39), higher practice (OR = 1.47; 1.35–1.60 per SD) and prior antibiotic prescribing (3+ vs. 1 prescriptions OR = 1.28; 1.04–1.57) and concurrent asthma medication (OR = 1.44; 1.32–1.57). Independent predictors of asthma medication in patients without asthma included higher prior asthma medication prescribing (≥7 vs. 0 prescriptions OR = 2.31; 1.83–2.91) and concurrent antibiotic prescribing (OR = 3.59; 3.22–4.01).

**Conclusion:**

Findings from the study indicate that antibiotics are over-used for ALRTI, irrespective of asthma status, and asthma medication is over-used in patients without asthma, with between-practice variation suggesting considerable clinical uncertainty. Further research is urgently needed to clarify the role of these medications for ALRTI.

## Background

Acute lower respiratory tract infections (ALRTI) is one of the most common acute illnesses managed in primary care, and accounts for between 8 and 10% of all primary care antibiotic prescribing [1]. In the UK, 63–70% of ALRTIs presenting at primary care are treated with antibiotics [2], despite good evidence they do not effectively reduce symptom duration or severity [3]. Prescribing unnecessary antibiotics not only contributes to a financial burden on health services, but also to antimicrobial resistance [4].

Asthma is one of the most common chronic diseases in the UK. For patients with existing asthma, ALRTIs can have a profound effect on the expression of disease. Viruses, most frequently Rhinovirus, are the predominant microorganisms associated with infective asthma exacerbations [5, 6]. The British Thoracic Society guidelines state that treatment of acute asthma exacerbations, even when infection is considered the trigger, should be steroids, and not antibiotics [7]. A recent Cochrane review found that using antibiotics for asthma exacerbation showed no reduction in symptoms [8]. However, the guidance on the management of ALRTIs in patients with asthma in primary care, with or without asthma exacerbation, remains unclear. Indeed, using clinical tools currently available it is not possible to establish if there is infection, inflammation or both present in these patients. Studies in children indicate that the simultaneous prescribing of antibiotics and asthma medications for respiratory tract infections is extremely common [9–11], but this has not been described in adults with asthma.

National antimicrobial resistance action plans aim to reduce the over-prescription and use of antibiotics. Recently, an increase in prescribing of asthma medication (including oral steroids) for patients without asthma has been reported [12, 13], despite good evidence that these are an ineffective treatment for ALRTI in these patients [14, 15].

In the UK, little has been published on the treatment of ALRTI by asthma status. The aim of the study was to describe the frequency and variation of antibiotic and asthma medication prescribing following a diagnosis for ALRTI, and investigate factors associated with prescribing among patients with and without asthma, using electronic health records in England.

## Method

### Study population

This was a retrospective cohort study using routinely collected, anonymised, primary care electronic health records of over 5 million active patients from around 650 UK general practices from the Clinical Practice Research Datalink (CPRD), considered representative of the general UK population [16]. The database includes the diagnostic codes used in routine clinical practice and all prescriptions. Approval was granted by the CPRD Independent Scientific Advisory Committee (protocol reference 16_290).

ALRTIs were identified using diagnostic codes for ALRTI (Additional file 1: Appendix 1) in patients aged 12 to 80 years presenting between 1 January 2014 and 31 December 2015. Patients aged < 12 years were excluded due to difficulties of reliably diagnosing asthma [17]. We also excluded patients for whom antibiotics were considered appropriate according to NICE guidelines, including those with chronic lung diseases other than asthma, e.g. chronic obstructive pulmonary disease [17]. The infection episode was the unit of analysis, thus a patient diagnosed with ≥1 ALRTI episode within the study period was included multiple times. Repeat recordings of ALRTI within 28 days were assumed to be a single episode, with the index date defined by the first code.

### Measurements

Current asthma status at ALRTI diagnosis was defined as ever having a recorded asthma diagnosis (including an asthma exacerbation code) and prescribed an asthma medication in the previous 12 months (Additional file 1: Appendices 2 and 3). Treatment for ALRTI was defined as a prescription for an orally administered antibiotic or a change in asthma medication prescribing within 3 days of diagnosis. Antibiotics included those most commonly used for ALRTI [18]: doxycycline, amoxicillin, clarithromycin, azithromycin, co-amoxiclav, erythromycin, tetracycline, cefalexin, and cefradine. A change in asthma medication was defined as an increase in dose, and/or an additional asthma medication prescribed, in relation to the previous month, for those with asthma, and a prescription for any asthma medication in patients without asthma.

Gender and age at first ALRTI diagnosis were calculated, and patients index of multiple deprivation (IMD) categorised by quintiles. Smoking status was ascertained using READ codes, and categorised as never, ex- and current smokers [19]. Season of infection was categorised as winter (1st January – 31st March 2014, 1st October 2014 – 31st March 2015, and 1st October – 31st December 2015) and summer. Number of ALRTI diagnoses, and all antibiotic (British National Formulary [BNF] chapter 5.1) and asthma medication (Additional file 1: Appendix 3) prescriptions in the 12 months prior to ALRTI diagnosis were derived.

A list of 37 physical and mental long-term conditions (Additional file 1: Appendix 4) established by clinical consensus were used to ascertain comorbidity status [20, 21]. A simple, unweighted count of clinical conditions was derived, and a four-category measure, grouping 0, 1, 2, and ≥ 3 conditions created.

Number of ALRTI episodes prescribed an antibiotic per 1000 population per year, using practice list size at the beginning of the study period, was calculated.

### Statistical analysis

Descriptive analysis was used to investigate differences in demographic and clinical factors, and ALRTI treatment, by asthma status. Variation in prescribing patterns (percentage of patients) across practices was explored using a mixed-effects model. The random effect for practice in a model with no covariates represents the unmeasured variation between practices.

Multi-level logistic regression models were fitted to investigate patient, clinical and practice factors associated with being prescribed an antibiotic or asthma medication for an ALRTI. Odd ratios (ORs) and 95% confidence intervals (CIs) were calculated. Univariate analysis of each variable was conducted, and measures with a *p*-value ≥0.25 were included in multivariate analysis (hereafter called the full model). Models were conducted in patients with and without asthma, separately.

Covariates were included as fixed effects, with a random intercept term for general practice allowing for within-practice comparisons. Thus, OR represents changes in odds of prescribing when covariates differ within the same practice. Age and practice ALRTI antibiotic rates were standardised using respective sample mean values and standard deviations (SD), and all continuous measurements were grouped into relevant categories, or quartiles. Non-linear associations were investigated in the full model, and the most appropriate, as determined using likelihood ratio tests, are presented.

Data analysis was conducted using STATA version 14 software [22]. All tests of significance were two-sided.

### Sensitivity analysis

To assess whether our asthma status definition affected prescribing estimates, particularly the use of asthma medication in patients without asthma, analyses were repeated using increasingly stringent definitions of non-asthma (previous asthma but no treatment within the last 5 years; and never having an asthma diagnosis). To evaluate whether the use of asthma medication in patients without asthma was an indication of an underlying asthma or COPD diagnosis, we repeated descriptive analysis excluding those with an asthma or COPD diagnosis code within a year of an infection. Finally, to assess whether patients with multiple infections influenced study findings, analyses were restricted to patients first infection during the study period. IMD was not available for 47.5% (*n* = 60,839) of ALRTI episodes, thus models were repeated without IMD, and findings compared.

## Results

During the study period, 127,976 ALRTIs were reported among 110,418 patients, of whom 17,952 (16.3%) had asthma (Table 1). Nearly all antibiotic and asthma medication (99.9%) were prescribed on the day of ALRTI diagnosis.

**Table 1:** Demographic and clinical characteristics of patients with at least one ALRTI^a^ in 2014–15, stratified by asthma status^b^

		Patients with asthma	Patients without asthma	*P*-value
*Demographic factors*				
Number of patients		17,952 (16.3)	92,466 (83.74)	
Gender, n (%)	Male	6391 (35.6)	38,509 (42.0)	
	Female	11,560 (64.4)	53,995 (58.4)	< 0.001
Age at ALRTI (years)	Mean (SD)	49.1 (17.3)	50.1 (17.3)	< 0.001
IMD quintile, n (%)	1 (Least)	1909 (20.5)	10,699 (21.8)	
	2	1862 (20.0)	10,133 (20.6)	
	3	1876 (20.2)	9746 (19.8)	
	4	1805 (19.4)	9164 (18.7)	
	5 (Most)	1849 (19.9)	9403 (19.1)	0.02
Smoking status, n (%)	Never smoker	6971 (38.8)	36,596 (39.6)	
	Smoker	7973 (44.4)	43,989 (47.6)	
	Previous	3008 (16.8)	11,875 (12.8)	< 0.001
*Clinical factors*				
Number of infections, n		20,990 (16.4)	106,986 (83.6)	
Season of infection	Summer	39,315 (36.8)	7648 (36.4)	
	Winter	67,671 (63.3)	13,342 (63.6)	0.39
Number (%) of ALRTI in prior 12 months	Mean (SD)	0.2 (0.5)	0.2 (0.5)	< 0.001
	0	17,737 (84.5)	93,279 (87.2)	
	1	2535 (12.1)	10,820 (10.1)	
	2+	718 (3.4)	2887 (2.7)	< 0.001
Multimorbidity score at index infection^c^, n (%)	Mean (SD)	1.7 (1.7)	1.4 (1.6)	< 0.001
	0	7003 (33.4)	43,529 (40.7)	
	1	4803 (22.9)	24,257 (22.7)	
	2	3484 (16.6)	16,575 (15.5)	
	3+	5700 (27.2)	22,625 (21.2)	< 0.001
Number (%) of asthma medications prescribed in previous 12 months^d^,	Mean (SD)	8.2 (8.5)	0.5 (2.5)	< 0.001
	0	–	101,210 (94.6)	
	1–6	11,797 (56.2)	5010 (4.7)	
	7+	9193 (43.8)	766 (0.7)	< 0.001
Number (%) of antibiotics prescribed in previous 12 months^e^	Mean (SD)	2.5 (2.8)	1.7 (2.0)	< 0.001
	0	2807 (13.4)	17,734 (16.6)	
	1	6471 (30.8)	48,091 (45)	
	2	4461 (21.3)	20,852 (19.5)	
	3+	7251 (34.6)	20,309 (19)	< 0.001
*Practice factors*				
Practice ALRTI antibiotic prescribing^f,^ per 1000 patients	Mean (SD)	801.6 (67.1)	798.0 (68.0)	< 0.001

*SD* Standard deviation, *IQR* Inter-quartile range, *IMD* Index multiple deprivation

Table 1 presents information from all ALRTI episodes. Demographic factor statistics are for patients (*n* = 110,418). Clinical factor statistics are for ALRTI episodes (*n* = 127,976). *P*-values report Pearson chi-squared test for independence for categorical variables and t-tests for equality of means for continuous measures.

^a^ Acute lower respiratory tract infection;

^b^ Asthma defined as ever diagnosed and prescribed an asthma medication in the 12 months prior to date of ALRTI

^c^ A list of 36 physical and mental chronic conditions (excluding asthma) were used to ascertain multimorbidity status in participants at ALRTI diagnosis [20, 23]

^d^ Asthma medication includes long- and short-acting β2 agonists, leukotriene receptor antagonists, antimuscarinic bronchodilators, and inhaled and oral corticosteroids

^e^ Any antibiotics (BNF chapter 5.1) prescribed in the last 12 months for any condition

^f^ Antibiotic prescribed within 3 days of a ALRTI diagnosis

Antibiotics were prescribed for nearly 80% of ALRTIs in both groups (Table 2), with amoxicillin and clarithromycin being the most common (70.6 and 13.3% of antibiotics prescribed, respectively). Asthma medication was prescribed in 41.1 and 14.7% of episodes (27 and 7% oral steroids) in patients with and without asthma, respectively. Among patients without asthma, salbutamol and prednisolone were the most commonly prescribed asthma medications (66.8 and 44.6% of asthma medications prescribed, respectively). For the majority (90.5%) of ALRTI episodes prescribed an asthma medication, patients were also given an antibiotic.

**Table 2:** Episode and measures of practice variance in medication prescribed within 3 days of ALRTI^a^ diagnosis, stratified by asthma^b^ status

	Patients with asthma	Patients without asthma
*ALRTI episodes, n = 127,976 (n, %)*		
No treatment	3374 (16.1)	20,293 (19.0)
Antibiotics only^c^	8985 (42.8)	70,944 (66.3)
^d^ Asthma medication only	508 (2.4)	1803 (1.7)
Oral steroid^e^	298 (1.4)	808 (0.8)
Antibiotics^c^ and asthma medication^d^	8123 (38.7)	13,946 (13.0)
Antibiotics^c^ and oral steroids^e^	5341 (25.4)	6318 (5.9)
*Practice variance, n = 513 (95% mid-range OR, 95% CI)* ^*f*^		
No treatment	3.09 (2.41, 4.23)	2.04 (1.81, 2.36)
Antibiotics only^c^	1.63 (1.46, 1.88)	1.89 (1.72, 2.13)
Asthma medication only^d^	7.46 (4.00, 18.46)	17.18 (10.08, 33.1)
Oral steroid^e^	17.76 (6.55, 81.91)	93.92 (33.15, 362.72)
Antibiotics^c^ and asthma medication^d^	1.62 (1.45, 1.87)	2.46 (2.14, 2.91)
Antibiotics^c^ and oral steroids^e^	2.94 (2.37, 3.84)	5.81 (4.39, 8.11)

*IQR* Interquartile range

^a^ Acute lower respiratory tract infection

^b^ Defined as ever diagnosed and asthma medication prescribed in the 12 months prior to ALRTI

^c^ Antibiotics limited to oral formulations and includes amoxicillin, doxycycline, clarithromycin, co-amoxiclav, azithromycin, erythromycin, tetracycline, cefalexin, cefradine

^d^ Among patients without asthma, asthma medication refers to any asthma medication, including oral corticosteroids. Among patients with asthma, asthma medication refers to an increase in the dose of current treatment and/or additional asthma medication (including oral corticosteroids) prescribed compared to the prior month

^e^ Oral steroids refer to oral corticosteroids

^f^ Practice variance was calculated from the variance of the random effect (σ2) and is given by e2 × 1.96 × σ and represents the odds ratio comparing a practice at the 2.5th percentile of the distribution of practices to one at the 97.5th percentile for the treatment outcome of interest. For example, amongst patients without asthma, practices who most frequently prescribed no treatment were 2.14 times more likely to prescribe no treatment compared to practices who prescribed no treatment the least

There was variation in the frequency of antibiotic and asthma medication prescribing across practices (Table 2). Overall, practices who prescribed antibiotics most frequently (97.5 percentile of antibiotics prescribing) were twice as likely to prescribe an antibiotic, compared to those who prescribed the least (2.5% percentile); odds ratio (OR) 1.92 (95% CI 1.69–2.26, *p* < 0.001) and 2.20 (1.92–2.58, *p* < 0.001) for patients with and without asthma, respectively. Practices at the top of the 95% mid-range of practices (i.e. 97.5th percentile compared to 2.5th percentile of asthma medication prescribing) had almost eight-times and 17-times the odds of prescribing an asthma medication only, compared to practices at the bottom, in patients with and without asthma, respectively.

### Predictors of antibiotic prescribing

In the full models, one of the strongest independent predictor of an antibiotic prescription, irrespective of asthma status, was the number of prior antibiotic prescriptions for any clinical indication: patients with 3 or more, compared to 1, prior antibiotic prescriptions were more likely to receive an antibiotic prescription; OR 1.28 (95% CI 1.04–1.57, *p* < 0.001) and 1.34 (1.21–1.49, *p* < 0.001) and in patients with and without asthma, respectively (Tables 3 and 4). A higher number of previous ALRTIs was inversely associated with antibiotic prescribing (≥2 vs. 0: 0.25; 0.16–0.39, *p* < 0.001; and 0.16; 0.13–0.20, *p* < 0.001, for patients with and without asthma, respectively), whilst increasing practice ALRTI antibiotic prescribing was positively associated with antibiotic prescribing (1.47; 1.35–1.60, *p* < 0.001; and 1.45; 1.39–1.52, *p* < 0.001 per SD for patients with and without asthma, respectively). Females were less likely to have an antibiotic prescription for an ALRTI, compared to males; 0.75 (0.63–0.90, *p* < 0.001) and 0.82 (0.76–0.88, *p* < 0.001), with and without asthma, respectively.

**Table 3:** Factors associated with an antibiotic prescription issued within 3 days of ALRTI^a^ episode in patients with asthma^b^

		No antibiotics, *n* (%)	Antibiotics, *n* (%)	Univariate analysis		Full model	
				OR (95% CI)	*p*-value	OR (95% CI)	*p*-value
		2052 (18.9)	8787 (81.1)				
*Demographic factors*							
Gender	Male	652 (31.8)	3127 (35.6)	1 (ref)		1 (ref)	
	Female	1400 (68.2)	5659 (64.4)	0.84 (0.76, 0.94)	< 0.001	0.75 (0.63, 0.90)	0.002
Age at ALRTI (years) ^c^	per SD			0.94 (0.89, 0.99)	0.01	1.02 (0.93, 1.12)	0.64
IMD quintile	1 (Least)	390 (19.0)	1765 (20.1)	1 (ref)		1 (ref)	
	2	363 (17.7)	1774 (20.2)	1.09 (0.92, 1.29)		1.27 (0.99, 1.65)	
	3	418 (20.4)	1733 (19.7)	0.94 (0.79, 1.11)		1.33 (1.02, 1.72)	
	4	424 (20.7)	1723 (19.6)	0.91 (0.76, 1.08)		1.26 (0.97, 1.64)	
	5 (Most)	457 (22.3)	1792 (20.4)	0.89 (0.74, 1.06)	0.17	1.52 (1.15, 1.99)	0.05
Current smoking status	Never	826 (40.3)	3415 (38.9)	1 (ref)		1 (ref)	
	Current	1007 (49.1)	3857 (43.9)	0.93 (0.83, 1.03)		1.04 (0.87, 1.25)	
	Previous	219 (10.7)	1515 (17.2)	1.71 (1.45, 2.02)	< 0.001	0.94 (0.74, 1.19)	0.69
*Clinical factors*							
Season of infection	Summer	786 (38.3)	3113 (35.4)	1 (ref)		1 (ref)	
	Winder	1266 (61.7)	5674 (64.6)	1.13 (1.02, 1.25)	0.02	1.14 (0.96, 1.34)	0.13
Prescribed asthma medication ^d^	No	1758 (85.7)	4600 (52.4)	1 (ref)		1 (ref)	
	Yes	294 (14.3)	4187 (47.7)	5.76 (5.04, 6.59)	< 0.001	1.44 (1.32, 1.57)	< 0.001
Number of ALRTI in prior 12 months	0	1214 (59.2)	7932 (90.3)	1 (ref)		1 (ref)	
	1	592 (28.9)	727 (8.3)	0.18 (0.16, 0.21)		0.52 (0.41, 0.67)	
	2+	246 (12.0)	128 (1.5)	0.07 (0.06, 0.09)	< 0.001	0.25 (0.17, 0.39)	< 0.001
Multimorbidity score ^e^	0	1135 (55.3)	2518 (28.7)	1 (ref)		1 (ref)	
	1	307 (15.0)	2279 (25.9)	3.34 (2.89, 3.85)		1.13 (0.90, 1.41)	
	2	221 (10.8)	1592 (18.1)	3.29 (2.80, 3.87)		1.35 (1.04, 1.76)	
	3+	389 (19.0)	2398 (27.3)	2.85 (2.49, 3.25)	< 0.001	1.05 (0.83, 1.33)	0.12
Number of antibiotics prescribed in previous 12 months ^f^	0	4045 (72.3)	116 (0.6)	0.001 (0.001, 0.002)		0.003 (0.002, 0.004)	
	1	600 (10.7)	7094 (33.5)	1 (ref)		1 (ref)	
	2	333 (6.0)	5290 (25.0)	1.26 (1.01, 1.57)		1.31 (1.05, 1.64)	
	3+	620 (11.1)	8705 (41.1)	1.06 (0.88, 1.28)	< 0.001	1.28 (1.04, 1.57)	< 0.001
*Practice factors*							
Practice ALRTI antibiotic rates (per 1000 patients) ^c^	per SD			1.55 (1.46, 1.64)	< 0.001	1.47 (1.35, 1.59)	< 0.001

*OR* Odds ratio, *CI* Confidence interval, *SD* Standard deviation, *ref* reference

Model restricted to participants with complete information on all variables included in the full model. Multi-level logistic regression models were used, with general practice included as a random effect to account for clustering. Coefficients represent the odds of receiving an antibiotic for a unit increase in the exposure of interest. Antibiotics prescribed within 3 days of ALRTI, and includes amoxicillin, doxycycline, clarithromycin, co-amoxiclav, azithromycin, erythromycin, tetracycline, cefalexin, and cefradine

^a^ Acute lower respiratory tract infection

^b^ Defined as ever diagnosed and asthma medication prescribed in the 12 months prior to ALRTI

^c^ Age at index infection and practice ALRTI antibiotic rates standardised (using sample mean values and SDs), and coefficients represent a change in the OR in antibiotic prescribing per 1-standard deviation increment in exposure of interest

^d^ Asthma medication refers to an increase in the dose of current treatment and/or additional asthma medication prescribed compared to the prior month for patients with asthma. Asthma medication includes long- and short-acting β2 agonists, leukotriene receptor antagonists, antimuscarinic bronchodilators, and inhaled and oral corticosteroids

^e^ A list of 37 physical and mental chronic conditions were used to ascertain multimorbidity status in participants at ALRTI diagnosis

^f^ Any antibiotics (BNF chapter 5.1) prescribed in the last 12 months for any condition

**Table 4:** Factors associated with an antibiotic prescription issued within 3 days of ALRTI^a^ episode in patients without asthma^b^

		No antibiotics, n (%)	Antibiotics, n (%)	Univariate analysis		Full model	
				OR (95% CI)	*p*-value	OR (95% CI)	*p*-value
		11,592 (20.1)	44,706 (79.4)				
*Demographic factors*							
Gender	Male	4339 (37.4)	18,589 (41.6)	1 (ref)		1 (ref)	
	Female	7253 (62.6)	26,117 (58.4)	0.84 (0.81, 0.88)	< 0.001	0.82 (0.76, 0.88)	< 0.001
Age at ALRTI (years) ^c^	per SD			0.90 (0.88, 0.92)	< 0.001	1.00 (0.96, 1.04)	0.87
IMD quintile	1 (Least)	2202 (19.0)	9737 (21.8)	1 (ref)		1 (ref)	
	2	2164 (18.7)	9275 (20.8)	0.95 (0.88, 1.02)		0.99 (0.88, 1.12)	
	3	2257 (19.5)	8824 (19.7)	0.87 (0.81, 0.94)		0.94 (0.83, 1.05)	
	4	2341 (20.2)	8321 (18.6)	0.79 (0.73, 0.85)		0.90 (0.80, 1.01)	
	5 (Most)	2628 (22.7)	8549 (19.1)	0.75 (0.69, 0.81)	< 0.001	1.01 (0.89, 1.15)	0.21
Smoking status	Never	4363 (37.6)	17,652 (39.5)	1 (ref)		1 (ref)	
	Current	6220 (53.7)	20,966 (46.9)	0.84 (0.81, 0.88)		1.00 (0.93, 1.08)	
	Previous	1009 (8.7)	6085 (13.6)	1.51 (1.40, 1.63)	< 0.001	1.19 (1.05, 1.34)	0.01
*Clinical factors*							
Season of infection	Summer	4391 (37.9)	15,994 (35.8)	1 (ref)		1 (ref)	
	Winder	7201 (62.1)	28,712 (64.2)	1.10 (1.05, 1.15)	< 0.001	1.06 (0.98, 1.14)	0.14
Prescribed asthma medication ^d^	No	10,548 (91.0)	37,436 (83.7)	1 (ref)		1 (ref)	
	Yes	1044 (9.0)	7270 (16.3)	2.11 (1.97, 2.27)	< 0.001	1.04 (0.94, 1.15)	0.43
Number of ALRTI in prior 12 months	0	7440 (64.2)	42,027 (94.0)	1 (ref)		1 (ref)	
	1	3131 (27.0)	2318 (5.2)	0.13 (0.12, 0.14)		0.24 (0.21, 0.27)	
	2+	1021 (8.8)	361 (0.8)	0.06 (0.05, 0.07)	< 0.001	0.16 (0.13, 0.20)	< 0.001
Multimorbidity score ^e^	0	6998 (60.4)	16,143 (36.1)	1 (ref)		1 (ref)	
	1	1626 (14.0)	11,484 (25.7)	3.14 (2.96, 3.33)		1.15 (1.05, 1.27)	
	2	1176 (10.1)	7513 (16.8)	2.87 (2.68, 3.08)		1.09 (0.97, 1.21)	
	3+	1792 (15.5)	9566 (21.4)	2.45 (2.31, 2.60)	< 0.001	0.96 (0.86, 1.07)	< 0.001
Number of antibiotics prescribed in previous 12 months ^f^	0	8636 (74.5)	532 (1.2)	0.004 (0.003, 0.004)		0.006 (0.005, 0.006)	
	1	1584 (13.7)	24,577 (55.0)	1 (ref)		1 (ref)	
	2	659 (5.7)	10,163 (22.7)	0.99 (0.90, 1.09)		1.16 (1.05, 1.28)	
	3+	713 (6.2)	9434 (21.1)	0.86 (0.78, 0.94)	< 0.001	1.35 (1.21, 1.49)	< 0.001
*Practice factors*							
Practice ALRTI antibiotic rates (per 1000 patients) ^c^	per SD			1.47 (1.44, 1.5)	< 0.001	1.45 (1.39, 1.52)	< 0.001

*OR* Odds ratio, *CI* Confidence interval, *SD* Standard deviation, *ref* reference

Models restricted to participants with complete information on all variables included in the full model. Multi-level logistic regression models were used, with general practice included as a random effect to account for clustering. Coefficients represent the odds of receiving antibiotic prescription for a unit increase in the exposure of interest. Antibiotics prescribed within 3 days of ALRTI, and includes amoxicillin, doxycycline, clarithromycin, co-amoxiclav, azithromycin, erythromycin, tetracycline, cefalexin, and cefradine

^a^ Acute lower respiratory tract infection

^b^ Defined as ever diagnosed and asthma medication prescribed in the 12 months prior to ALRTI

^c^ Age at index infection and practice ALRTI antibiotic rates standardised (using sample mean values and SDs), and coefficients represent a change in the OR in antibiotic prescribing per 1-standard deviation increment in exposure of interest

^d^ Asthma medication refers to a prescription for any asthma medication. Asthma medication includes long- and short-acting β2 agonists, leukotriene receptor antagonists, antimuscarinic bronchodilators, and inhaled and oral corticosteroids

^e^ A list of 37 physical and mental chronic conditions were used to ascertain multimorbidity status in participants at ALRTI diagnosis

^f^ Any antibiotics (BNF chapter 5.1) prescribed in the last 12 months for any condition

Among patients with asthma, a concurrent change in asthma medication and IMD quintile, previous smoking status and multimorbidity (Table 4), were also positively associated with receiving an antibiotic. Age and season of infection were not independently associated with an antibiotic prescription. Smoking status and multimorbidity in patients with asthma (Table 3), and IMD quintile and asthma medication in patients without asthma (Table 4) were not independently associated with an antibiotic prescription.

### Predictors of asthma medication prescribing

In the full models (Tables 5 and 6), a corresponding antibiotic prescription was the strongest independent predictor of a change in asthma medication (5.22; 4.54–6.01, *p* < 0.001 and 3.59; 3.22–4.01, *p* < 0.001 for patients with and without asthma, respectively). Prior prescriptions for asthma medication were also positively associated with a prescription; patients with asthma 2–4 vs. 1 prescription 1.14; 1.01–1.29, *p* < 0.001; and patients without asthma ≥7 vs. 0 prescriptions 2.31; 1.83–2.91, *p* < 0.001). Other predictors of being prescribed an asthma medication included a positive association with current smoking status, inverse relationship with age and number of prior ALRTI, and mixed (with asthma) and positive (without asthma) association with multimorbidity.

**Table 5:** Factors associated with a change in asthma medication within 3 days of ALRTI^a^ episode in patients with asthma^b^

		No asthma medication	Asthma medication	Univariate analysis		Full model	
				OR (95% CI)	*p*-value	OR (95% CI)	*p*-value
		6358 (58.7)	4481 (41.3)				
*Demographic factors*							
Gender	Male	2187 (34.4)	1592 (35.5)	1 (ref)			
	Female	4170 (65.6)	2889 (64.5)	0.95 (0.87, 1.03)	0.21		
Age at ALRTI (years)	Q1 (< 39)	1477 (23.2)	1219 (27.2)	1 (ref)		1 (ref)	
	Q2 (39-)	1534 (24.1)	1198 (26.7)	0.93 (0.84, 1.04)		0.97 (0.87, 1.09)	
	Q3 (52-)	1620 (25.5)	1055 (23.5)	0.77 (0.69, 0.87)		0.85 (0.75, 0.96)	
	Q4 (64+)	1727 (27.2)	1009 (22.5)	0.70 (0.62, 0.78)	< 0.001	0.75 (0.66, 0.85)	< 0.001
IMD quintile	1 (Least)	1249 (19.6)	906 (20.2)	1 (ref)			
	2	1277 (20.1)	860 (19.2)	0.94 (0.83, 1.07)			
	3	1215 (19.1)	936 (20.9)	1.06 (0.93, 1.21)			
	4	1271 (20.0)	876 (19.6)	0.95 (0.83, 1.09)			
	5 (Most)	1346 (21.2)	903 (20.2)	0.95 (0.82, 1.09)	0.31		
Smoking status	Never	2539 (39.9)	1702 (38.0)	1 (ref)		1 (ref)	
	Current	2815 (44.3)	2049 (45.7)	1.09 (1.00, 1.19)		1.13 (1.03, 1.24)	
	Previous	1004 (15.8)	730 (16.3)	1.09 (0.97, 1.22)	0.13	1.04 (0.92, 1.17)	0.03
*Clinical factors*							
Season of infection	Summer	2261 (35.6)	1638 (36.6)	1 (ref)			
	Winder	4097 (64.4)	2843 (63.5)	0.96 (0.89, 1.04)	0.37		
Prescribed an antibiotic ^c^	No	1758 (27.7)	294 (6.6)	1 (ref)		1 (ref)	
	Yes	4600 (72.4)	4187 (93.4)	5.69 (4.98, 6.50)	< 0.001	5.22 (4.54, 6.01)	< 0.001
Number of ALRTI in prior 12 months	0	5114 (80.4)	4032 (90.0)	1 (ref)		1 (ref)	
	1	935 (14.7)	384 (8.6)	0.53 (0.46, 0.60)		0.81 (0.71, 0.93)	
	2+	309 (4.9)	65 (1.5)	0.27 (0.21, 0.36)	< 0.001	0.57 (0.42, 0.76)	< 0.001
Multimorbidity score ^d^	0	2249 (35.4)	1404 (31.3)	1 (ref)		1 (ref)	
	1	1386 (21.8)	1200 (26.8)	1.38 (1.25, 1.53)		1.10 (0.98, 1.22)	
	2	1017 (16.0)	796 (17.8)	1.25 (1.11, 1.40)		1.03 (0.90, 1.16)	
	3+	1706 (26.8)	1081 (24.1)	1.00 (0.90, 1.11)	< 0.001	0.88 (0.78, 0.99)	< 0.001
Number of asthma medications prescribed in previous 12 months	Q1 (1)	1158 (18.2)	700 (15.6)	1 (ref)		1 (ref)	
	Q2 (2-)	2057 (32.4)	1691 (37.7)	1.35 (1.20, 1.51)		1.14 (1.01, 1.29)	
	Q3 (5-)	1406 (22.1)	1014 (22.6)	1.18 (1.04, 1.34)		1.03 (0.90, 1.17)	
	Q4 (11+)	1737 (27.3)	1076 (24.0)	1.01 (0.90, 1.15)	< 0.001	0.96 (0.84, 1.09)	0.01

*OR* odds ratio; *CI* confidence interval; *ref* reference; *Q* quartile

Models restricted to participants with complete information on all variables included in the full model. Multi-level logistic regression models were used, with general practice included as a random effect to account for clustering. Coefficients represent the odds of receiving asthma medication for a unit increase in the exposure of interest. IMD quintile and gender were excluded from the analysis as the model did not converge. Asthma medication refers to a an increase in the dose of current treatment and/or additional asthma medication prescribed compared to the prior month. Asthma medication includes long- and short-acting β2 agonists, leukotriene receptor antagonists, antimuscarinic bronchodilators, and inhaled and oral corticosteroids

^a^ Acute lower respiratory tract infection

^b^ Defined as ever diagnosed and asthma medication prescribed in the 12 months prior to ALRTI

^c^ Antibiotics prescribed within 3 days of ALRTI, and includes amoxicillin, doxycycline, clarithromycin, co-amoxiclav, azithromycin, erythromycin, tetracycline, cefalexin, and cefradine

^d^ A list of 37 physical and mental chronic conditions were used to ascertain multimorbidity status in participants at ALRTI diagnosis

**Table 6:** Factors associated with asthma medication within 3 days of ALRTI^a^ episode in patients without asthma^b^

		No asthma medication	Asthma medication	Univariate analysis		Full model	
				OR (95% CI)	*p*-value	OR (95% CI)	*p*-value
		47,984 (85.2)	8314 (14.8)				
*Demographic factors*							
Gender	Male	19,910 (41.5)	3018 (36.3)	1 (ref)		1 (ref)	
	Female	28,074 (58.5)	5296 (63.7)	1.25 (1.19, 1.31)	< 0.001	1.10 (1.03, 1.18)	0.01
Age at ALRTI (years)	Q1 (< 39)	11,264 (23.5)	2377 (28.6)	1 (ref)		1 (ref)	
	Q2 (39-)	11,815 (24.6)	2319 (27.9)	0.92 (0.87, 0.99)		1.02 (0.93, 1.12)	
	Q3 (52-)	12,583 (26.2)	2067 (24.9)	0.78 (0.73, 0.83)		0.81 (0.73, 0.89)	
	Q4 (64+)	12,322 (25.7)	1551 (18.7)	0.60 (0.56, 0.65)	< 0.001	0.59 (0.53, 0.66)	< 0.001
IMD quintile	1 (Least)	10,272 (21.4)	1667 (20.1)	1 (ref)		1 (ref)	
	2	9856 (20.5)	1583 (19.0)	0.98 (0.91, 1.07)		0.92 (0.82, 1.03)	
	3	9371 (19.5)	1710 (20.6)	1.08 (0.99, 1.17)		1.03 (0.91, 1.15)	
	4	9027 (18.8)	1635 (19.7)	1.12 (1.03, 1.23)		0.94 (0.84, 1.07)	
	5 (Most)	9458 (19.7)	1719 (20.7)	1.20 (1.09, 1.32)	< 0.001	0.97 (0.86, 1.11)	0.31
Smoking status	Never	19,115 (39.8)	2900 (34.9)	1 (ref)		1 (ref)	
	Current	22,726 (47.4)	4460 (53.7)	1.30 (1.23, 1.36)		1.10 (1.02, 1.19)	
	Previous	6141 (12.8)	953 (11.5)	1.02 (0.94, 1.10)	< 0.001	1.00 (0.89, 1.12)	0.02
*Clinical factors*							
*Season of infection*	Summer	17,348 (36.2)	3037 (36.5)	1 (ref)			
	Winter	30,636 (63.9)	5277 (63.5)	0.98 (0.93, 1.03)	0.47		
Prescribed an oral antibiotic ^c^	No	10,548 (22.0)	1044 (12.6)	1 (ref)		1 (ref)	
	Yes	37,436 (78.0)	7270 (87.4)	2.11 (1.97, 2.27)		3.59 (3.22, 4.01)	
Number of ALRTI in prior 12 months, per ALRTI				0.85 (0.81, 0.90)	< 0.001	0.89 (0.83, 0.97)	0.01
Multimorbidity score ^d^	0	20,119 (41.9)	3022 (36.4)	1 (ref)		1 (ref)	
	1	10,960 (22.8)	2150 (25.9)	1.30 (1.22, 1.38)		1.15 (1.05, 1.26)	
	2	7328 (15.3)	1361 (16.4)	1.25 (1.16, 1.34)		1.07 (0.96, 1.19)	
	3+	9577 (20.0)	1781 (21.4)	1.24 (1.16, 1.32)	< 0.001	1.08 (0.98, 1.19)	0.02
Number of asthma medications prescribed in previous 12 months	0	13,486 (86.6)	3813 (80.2)	1 (ref)		1 (ref)	
	1–6	1867 (12)	808 (17.0)	1.50 (1.37, 1.65)		1.57 (1.43, 1.73)	
	7+	221 (1.4)	131 (2.8)	2.18 (1.74, 2.74)	< 0.001	2.31 (1.83, 2.91)	< 0.001

*OR* odds ratio; *CI* confidence interval; *ref* reference; *Q* quartile

Models restricted to participants with complete information on all variables included in the full model. Multi-level logistic regression models were used, with general practice included as a random effect to account for clustering. Coefficients represent the odds of receiving asthma medication for a unit increase in the exposure of interest. Asthma medication refers to any asthma medication, including long- and short-acting β2 agonists, leukotriene receptor antagonists, antimuscarinic bronchodilators, and inhaled and oral corticosteroids

^a^ Acute lower respiratory tract infection

^b^ Defined as ever diagnosed and asthma medication prescribed in the 12 months prior to ALRTI

^c^ Antibiotics prescribed within 3 days of ALRTI, and includes amoxicillin, doxycycline, clarithromycin, co-amoxiclav, azithromycin, erythromycin, tetracycline, cefalexin, and cefradine

^d^ A list of 37 physical and mental chronic conditions were used to ascertain multimorbidity status in participants at ALRTI diagnosis

Among patients without asthma, females, compared to males, were more likely to receive a prescription for asthma medication (Table 5).

IMD quintile and season of infection were not associated with an asthma medication prescription. Among those with asthma, gender was not associated with a change in asthma medication (Table 6).

### Sensitivity analysis

Overall, broadening our asthma definition status made little difference to general prescribing trends across groups (Additional file 1: Appendix 5). Among patients ever diagnosed with asthma, 73.5% of ALRTI episodes were prescribed an antibiotic (compared to 81.5% in the main analysis) and 33.9% had a change in asthma medication (compared to 41.1%). Among those who had never had an asthma diagnosis, 82.4 and 12.3% of ALRTI episodes were prescribed an antibiotic and asthma medication, respectively (compared to 79.3 and 14.7%, respectively in the main analysis). Likewise, excluding patients without asthma who were diagnosed with asthma or COPD within a year of an index infection had little impact on the results. Asthma medications were prescribed for 14.3% of patients without asthma in sensitivity analysis, compared to 14.7% in the main analysis (data not shown).

To assess the influence of multiple infections, sensitivity analysis restricted to the first ALRTI episode was performed (Additional file 1: Appendix 6). Prior antibiotic prescribing for any clinical indication was not positively associated with antibiotic prescribing in sensitivity analysis, due to more patients having an antibiotic prescription in the prior 12 months for later infection episodes, compared to the first ALRTI episode, especially for those prescribed antibiotics (51.5% vs. 18.3%). Unlike in the main analysis, where an inverse relationship was observed between number of prior ALRTIs and antibiotics in patients with asthma, and asthma medication in patients without asthma, no relationship was found in the sensitivity analysis.

No differences were observed in models where IMD was excluded (data not shown).

## Discussion

### Summary

The main findings from this investigation of routine health records in a patient population at low-risk of pneumonia suggest that antibiotics were frequently prescribed for ALRTIs irrespective of asthma status, in contrast to NICE recommendations [24]. Changes to asthma medication prescriptions was observed in only 41% of ALRTI episodes in patients with asthma, but higher than expected levels were found in patients without asthma at 15%, with 40% of these including oral corticosteroids. Asthma medication was infrequently prescribed without antibiotics. The considerable variation in prescribing across practices, especially for asthma medication, highlights the clinical uncertainty in treating ALRTI. Factors associated with prescribing of both antibiotics and asthma medication were broadly similar irrespective of asthma status, with the strongest factors relating to the frequency of previous ALRTI episodes and treatment.

### Strengths and limitations

To our knowledge, this is the first study to use routine medical records to investigate antibiotic and asthma medication use for ALRTIs by asthma status in patients at low risk of pneumonia. The prescribing data are reliable since electronic prescribing is ubiquitous in UK primary care, and similarly, the coding of factors placing patients at high risk of pneumonia, and most patient demographics are reliably coded.

Despite these strengths, there are limitations. First, the relationship between ALRTI, and antibiotic and asthma medication prescribing, is temporal. Although likely to be linked, in some cases medication could have been prescribed for other reasons. However, most medications were prescribed on the day of ALRTI diagnosis. Second, CPRD is a database of electronic medical records, and coding quality may influence study findings, particularly diagnostic codes, with a clinicians decision to record a diagnosis potentially linked to prescribing choices [25]. However, we used a broad range of clinical codes to define and capture ALRTIs and findings of high-levels of antibiotic use are in keeping with previous general population studies that have used clinical inclusion criteria [26, 27]. Third, clinician’s often advice patients with asthma to increase the dose of existing asthma medications, and thus an analysis based on a change in prescription will probably under-estimate a change in use of asthma medication. Finally, our definition of asthma may mean some patients with a diagnosis of asthma will be in the non-asthma group potentially leading to an over-estimation of asthma medication in this population. Clinicians who suspect asthma or COPD may also be more likely to prescribe an asthma medication without clinically diagnosing or reporting. However, we used a standard method which has been shown to accurately reflect asthma status in CPRD [28], and explored broader definitions and excluded patients later diagnosed with asthma and COPD in sensitivity analysis. Furthermore, the prevalence of asthma was comparable to the UK lifetime prevalence in a large, linked database study (16.3% vs. 15.6%) [29].

### Comparisons with existing literature

Our finding of high levels of antibiotic prescribing for ALRTI in primary care are consistent with the established literature [27, 30, 31]. Results indicate that there is little difference in prescribing of antibiotics by asthma status, despite guidance that they should be avoided [24], and evidence that they are not effective in treating ALRTI [8]. There has been little investigation of antibiotic prescribing for ALRTI in adults with asthma, although the available evidence indicates that antibiotics are still frequently prescribed for asthma exacerbations [32, 33].

Antibiotic prescribing was associated with patients’ prior patterns of antibiotic prescribing, although when analysis was restricted to the first ALRTI episode, this association was attenuated in patients with asthma and reversed in those without. Results indicate that prior prescribing influences the likelihood of being prescribed an antibiotic for subsequent presentations, with infrequent prescribing in patients not prescribed antibiotics in the recent past. Prescribing practices are likely to be influenced by doctor and practice characteristics as well as patient expectations, as shown in other studies [34, 35]. Practice ALRTI prescribing was positively associated with antibiotic use, comparable to an earlier analysis of UK primary care data [30].

A surprising finding was the negative association between antibiotic prescribing and frequent ALRTI episodes in the patients recent past. This may be due to characteristics associated with frequent attenders, or indicate GPs reluctance to provide an antibiotic prescription multiple times for the same indication. Indeed, of those patients diagnosed with two or more ALRTI episodes (3605), two-thirds (65.6%) had not had an antibiotic prescription in the same period, compared to 10.5% of patients who had not had a prior ALRTI episode, suggesting clinicians were more likely to prescribe antibiotics to patients who don’t often present, perhaps because they had a more severe illness. A further unexpected result was the inverse correlation between multimorbidity score and an antibiotic prescription in patients without asthma, and a change in asthma medication among patients with asthma. Patients with multiple chronic conditions are seen more frequently by the GP and therefore, clinicians may be more inclined to delay prescribing. Age was also inversely associated with an asthma medication, indicating clinicians were less likely to increase the dose of asthma medication for elderly, multimorbid patients.

Asthma medication was used frequently, irrespective of asthma status, and commonly alongside an antibiotic. International studies have also found antibiotics and corticosteroids are commonly co-prescribed for asthma exacerbations [32], and in patients without asthma [36, 37]. Indeed, here, an antibiotic prescription was one of the strongest predictors of being prescribed an asthma medication.

### Implications for research and/or practice

Our study provides evidence that general practices are continuing to frequently prescribe antibiotics for ALRTI, despite evidence of limited benefit, and in contradiction to national guidelines. Furthermore, practitioners frequently co-prescribe asthma medication and antibiotics, which may reflect uncertainty regarding the underlying aetiology*.* High quality research has been conducted to determine the lack of effectiveness of antibiotics in ALRTI in a non-asthmatic low-risk patient population [3] and in reducing symptoms of asthma exacerbations [8], but research is now needed to address the clinical uncertainty in the optimum management of ALRTI in patients with asthma.

## Conclusion

We have demonstrated high-use of antibiotics and asthma medication for the treatment of ALRTI in patients with and without asthma, respectively, with considerable between-practice variation. Further research is urgently needed to inform optimum use of both antibiotics and asthma medication for patients with ALRTI.

## Supplementary information

**Additional file 1:** **Appendix 1.** Acute lower respiratory tract infection medical codes. **Appendix 2.** Asthma status medical codes. **Appendix 3.** Asthma status treatment product codes. **Appendix 4.** Description of conditions and definitions of chronic conditions included in the multimorbidity score. **Appendix 5.** Episode and measures of practice variance in medication prescribed within three days of ALRTIa diagnosis. **Appendix 6.** Sensitivity analysis: multivariate final models investigating factors associated with an antibiotic prescription or change in asthma medication within three days of an ALRTIa episode, stratified by asthma statusb and restricted to patients first ALRTI infection in the study period.

## Data Availability

The data that support the findings of this study are available from CPRD but restrictions apply to the availability of these data, which were used under license for the current study, and so are not publicly available. The data used in the final analysis are however available from the authors upon reasonable request and with permission of CPRD.

## Abbreviations

ALRTI: Acute lower respiratory tract infections
BNF: British national formulary
CI: Confidence intervals
CPRD: Clinical practice research datalink
IMD: Index of multiple deprivation
OR: Odds ratio
SD: Standard deviation
