# Supplementary material for: Use of antibiotics and asthma medication for acute lower respiratory tract infections in people with and without asthma: retrospective cohort study
Source: Respir Res. 2020 Jan 6;21:4. doi: 10.1186/s12931-019-1233-5 (PMC6945474; doi:10.1186/s12931-019-1233-5)
Supplement: Supplementary file 1 — Additional file 1: Appendix 1. Acute lower respiratory tract infection medical codes. Appendix 2. Asthma status medical codes. Appendix 3. Asthma status treatment product codes. Appendix 4. Description of conditions and definitions of chronic conditions included in the multimorbidity score. Appendix 5. Episode and measures of practice variance in medication prescribed within three days of ALRTIa diagnosis. Appendix 6. Sensitivity analysis: multivariate final models investigating factors associated with an antibiotic prescription or change in asthma medication within three days of an ALRTIa episode, stratified by asthma statusb and restricted to patients first ALRTI infection in the study period. [file 12931_2019_1233_MOESM1_ESM.docx]

Appendix1: Acute lower respiratory tract infection medical codes

| **READ code** | **READ term** | **Events** |
| --- | --- | --- |
| H06z000 | Chest infection NOS | 77783 |
| H06z011 | Chest infection | 46279 |
| H060.00 | Acute bronchitis | 14867 |
| H062.00 | Acute lower respiratory tract infection | 7482 |
| H060.11 | Acute wheezy bronchitis | 2088 |
| H302.00 | Wheezy bronchitis | 414 |
| H06z200 | Recurrent chest infection | 274 |
| H06..00 | Acute bronchitis and bronchiolitis | 251 |
| H060w00 | Acute viral bronchitis unspecified | 238 |
| H300.00 | Tracheobronchitis NOS | 71 |
| H060z00 | Acute bronchitis NOS | 64 |
| H060500 | Acute tracheobronchitis | 46 |
| H06z112 | Acute lower respiratory tract infection | 21 |
| H301.00 | Laryngotracheobronchitis | 19 |
| H30..11 | Chest infection - unspecified bronchitis | 16 |
| Hyu1.00 | [X]Other acute lower respiratory infections | 2 |
| H06z.00 | Acute bronchitis or bronchiolitis NOS | 2 |
| H060600 | Acute pneumococcal bronchitis | 1 |
| H060300 | Acute purulent bronchitis | 1 |
| H060F00 | Acute bronchitis due to echovirus |  |
| H060D00 | Acute bronchitis due to respiratory syncytial virus |  |
| H060v00 | Subacute bronchitis unspecified |  |
| Hyu1000 | [X]Acute bronchitis due to other specified organisms |  |
| H060C00 | Acute bronchitis due to parainfluenza virus |  |
| H460000 | Acute bronchitis due to chemical fumes |  |
| H060B00 | Acute bronchitis due to coxsackievirus |  |
| H060E00 | Acute bronchitis due to rhinovirus |  |
| H060x00 | Acute bacterial bronchitis unspecified |  |
| H060400 | Acute croupous bronchitis |  |
| H060000 | Acute fibrinous bronchitis |  |
| H060800 | Acute haemophilus influenzae bronchitis |  |
| H060100 | Acute membranous bronchitis |  |
| H060200 | Acute pseudomembranous bronchitis |  |
| H060700 | Acute streptococcal bronchitis |  |
| H24..11 | Chest infection with infectious disease EC |  |

Events greater than the number of episodes in analysis (n=128,976) due to multiple codes used for one episode

Appendix 2: Asthma status medical codes

| **READ code** | **READ term** | **READ code** | **READ term** |
| --- | --- | --- | --- |
| H33..00 | Asthma | 1O2..00 | Asthma confirmed |
| H333.00 | Acute exacerbation of asthma | H33z200 | Late-onset asthma |
| H33z100 | Asthma attack | 663V200 | Moderate asthma |
| H33z011 | Severe asthma attack | H330000 | Extrinsic asthma without status asthmaticus |
| H330.12 | Childhood asthma | H330.13 | Hay fever with asthma |
| H33..11 | Bronchial asthma | H33zz00 | Asthma NOS |
| H330.11 | Allergic asthma | H33zz13 | Allergic bronchitis NEC |
| 663V100 | Mild asthma | H331111 | Intrinsic asthma with asthma attack |
| 663V300 | Severe asthma | H33zz12 | Allergic asthma NEC |
| 663V000 | Occasional asthma | 173c.00 | Occupational asthma |
| H331.11 | Late onset asthma | G581.11 | Asthma - cardiac |
| H33z.00 | Asthma unspecified | H332.00 | Mixed asthma |
| H33zz11 | Exercise induced asthma | H330100 | Extrinsic asthma with status asthmaticus |
| H33z000 | Status asthmaticus NOS | H331000 | Intrinsic asthma without status asthmaticus |
| H331.00 | Intrinsic asthma | H33z.11 | Hyperreactive airways disease |
| H330011 | Hay fever with asthma | H35y700 | Wood asthma |
| H312000 | Chronic asthmatic bronchitis | H334.00 | Brittle asthma |
| 173A.00 | Exercise induced asthma | H331z00 | Intrinsic asthma NOS |
| H330111 | Extrinsic asthma with asthma attack | H330z00 | Extrinsic asthma NOS |
| 8H2P.00 | Emergency admission, asthma | H47y000 | Detergent asthma |
| H330.00 | Extrinsic (atopic) asthma | H331100 | Intrinsic asthma with status asthmaticus |
| H330.14 | Pollen asthma | 173d.00 | Work aggravated asthma |
| H33z111 | Asthma attack NOS | H35y600 | Sequoiosis (red-cedar asthma) |
| 663j.00 | Asthma - currently active | H335.00 | Chronic asthma with fixed airflow obstruction |

Appendix 3: Asthma status treatment product codes

| **Product code** | **Product name** | **Drug substance** | **Oral steroid** |
| --- | --- | --- | --- |
| 24023 | Theodrox Tablet (3M Health Care Ltd) | Aluminium Hydroxide/Aminophylline | 1 |
| 555 | Aminophylline 225mg modified-release tablets | Aminophylline | 1 |
| 8056 | Aminophylline 100mg tablets | Aminophylline | 1 |
| 10561 | Aminophylline 250mg/ml injection | Aminophylline |  |
| 12699 | Pecram 225mg Modified-release tablet (Novartis Consumer Health UK Ltd) | Aminophylline | 1 |
| 13529 | Amnivent-225 SR tablets (Ashbourne Pharmaceuticals Ltd) | Aminophylline | 1 |
| 14739 | Norphyllin SR 225mg tablets (Teva UK Ltd) | Aminophylline | 1 |
| 14991 | Aminophylline 250mg/10ml injection | Aminophylline |  |
| 17140 | Aminophylline 200mg tablets | Aminophylline | 1 |
| 23572 | Aminophylline sr 225mg Modified-release tablet (IVAX Pharmaceuticals UK Ltd) | Aminophylline | 1 |
| 25125 | Aminophylline 360mg suppositories | Aminophylline |  |
| 28241 | Aminophylline 250mg/10ml solution for injection Minijet pre-filled syringes (UCB Pharma Ltd) | Aminophylline | 1 |
| 29273 | Aminophylline 225mg Modified-release tablet (Hillcross Pharmaceuticals Ltd) | Aminophylline | 1 |
| 30596 | Aminophylline 225mg Modified-release tablet (Actavis UK Ltd) | Aminophylline | 1 |
| 42511 | Aminophylline 25mg/ml Injection (Celltech Pharma Europe Ltd) | Aminophylline |  |
| 42910 | Aminophylline 250mg/10ml solution for injection ampoules (Martindale Pharmaceuticals Ltd) | Aminophylline |  |
| 590 | Phyllocontin Continus 225mg tablets (Napp Pharmaceuticals Ltd) | Aminophylline hydrate | 1 |
| 4514 | Aminophylline 350mg modified-release tablets | Aminophylline Hydrate | 1 |
| 6988 | Aminophylline hydrate 100mg modified-release tablets | Aminophylline Hydrate | 1 |
| 8057 | Aminophylline 100mg modified-release tablets | Aminophylline Hydrate | 1 |
| 8806 | Phyllocontin continus 350mg Tablet (Napp Pharmaceuticals Ltd) | Aminophylline hydrate | 1 |
| 10407 | Phyllocontin Paediatric Continus 100mg tablets (Napp Pharmaceuticals Ltd) | Aminophylline hydrate | 1 |
| 16994 | Aminophylline hydrate 350mg modified-release tablets | Aminophylline hydrate | 1 |
| 17002 | Aminophylline hydrate 225mg modified-release tablets | Aminophylline hydrate | 1 |
| 7192 | Bambuterol 10mg tablets | Bambuterol hydrochloride | 1 |
| 12144 | Bambuterol 20mg tablets | Bambuterol hydrochloride | 1 |
| 13575 | Bambec 20mg tablets (AstraZeneca UK Ltd) | Bambuterol hydrochloride | 1 |
| 14527 | Bambec 10mg tablets (AstraZeneca UK Ltd) | Bambuterol hydrochloride | 1 |
| 38 | Beclometasone 100micrograms/dose inhaler | Beclometasone dipropionate |  |
| 99 | Becotide 100 inhaler (GlaxoSmithKline UK Ltd) | Beclometasone dipropionate |  |
| 883 | Becodisks 200microgram Disc (Allen & Hanburys Ltd) | Beclometasone Dipropionate |  |
| 895 | Beclazone 100 Easi-Breathe inhaler (Teva UK Ltd) | Beclometasone dipropionate |  |
| 896 | Becotide easi-breathe 100microgram/actuation Pressurised inhalation (Allen & Hanburys Ltd) | Beclometasone dipropionate |  |
| 1100 | Beclazone 100 inhaler (Teva UK Ltd) | Beclometasone dipropionate |  |
| 1236 | Becloforte 250micrograms/dose inhaler (GlaxoSmithKline UK Ltd) | Beclometasone dipropionate |  |
| 1242 | Beclometasone 250micrograms/dose inhaler | Beclometasone dipropionate |  |
| 1243 | Beclazone 250 Easi-Breathe inhaler (Teva UK Ltd) | Beclometasone dipropionate |  |
| 1258 | Becotide 200 inhaler (GlaxoSmithKline UK Ltd) | Beclometasone dipropionate |  |
| 1259 | Beclometasone 200micrograms/dose inhaler | Beclometasone dipropionate |  |
| 1269 | Becotide 50microgram/ml Nebuliser liquid (Allen & Hanburys Ltd) | Beclometasone Dipropionate |  |
| 1406 | Becotide 50 inhaler (GlaxoSmithKline UK Ltd) | Beclometasone dipropionate |  |
| 1537 | Becotide 200microgram Rotacaps (GlaxoSmithKline UK Ltd) | Beclometasone dipropionate |  |
| 1551 | Beclazone 250 inhaler (Teva UK Ltd) | Beclometasone dipropionate |  |
| 1552 | Becloforte easi-breathe 250microgram/actuation Pressurised inhalation (Allen & Hanburys Ltd) | Beclometasone dipropionate |  |
| 1725 | Beclazone 50 Easi-Breathe inhaler (Teva UK Ltd) | Beclometasone dipropionate |  |
| 1727 | Becotide easi-breathe 50microgram/actuation Pressurised inhalation (Allen & Hanburys Ltd) | Beclometasone dipropionate |  |
| 1734 | Beclometasone 100micrograms/dose breath actuated inhaler | Beclometasone dipropionate |  |
| 1861 | AeroBec 100 Autohaler (Meda Pharmaceuticals Ltd) | Beclometasone dipropionate |  |
| 1885 | Beclazone 200 inhaler (Teva UK Ltd) | Beclometasone dipropionate |  |
| 1951 | Becodisks 400microgram Disc (Allen & Hanburys Ltd) | Beclometasone Dipropionate |  |
| 2148 | Beclometasone 400microgram disc | Beclometasone Dipropionate |  |
| 2159 | AeroBec 50 Autohaler (Meda Pharmaceuticals Ltd) | Beclometasone dipropionate |  |
| 2160 | Beclometasone 50micrograms/dose breath actuated inhaler | Beclometasone dipropionate |  |
| 2229 | Becodisks 100microgram Disc (Allen & Hanburys Ltd) | Beclometasone Dipropionate |  |
| 2335 | Qvar 100 inhaler (Teva UK Ltd) | Beclometasone dipropionate |  |
| 2600 | Beclometasone 250micrograms/dose breath actuated inhaler | Beclometasone dipropionate |  |
| 2892 | Becloforte 400microgram disks (GlaxoSmithKline UK Ltd) | Beclometasone dipropionate |  |
| 2893 | Beclometasone 200micrograms disc | Beclometasone Dipropionate |  |
| 2992 | Beclazone 50 inhaler (Teva UK Ltd) | Beclometasone dipropionate |  |
| 3018 | Beclometasone 50micrograms/dose inhaler | Beclometasone dipropionate |  |
| 3075 | Becotide 400microgram Rotacaps (GlaxoSmithKline UK Ltd) | Beclometasone dipropionate |  |
| 3119 | Becloforte integra 250microgram/actuation Inhaler with compact spacer (Glaxo Laboratories Ltd) | Beclometasone Dipropionate |  |
| 3150 | Beclometasone 100micrograms/actuation extrafine particle cfc free inhaler | Beclometasone Dipropionate |  |
| 3220 | Qvar 50 Autohaler (Teva UK Ltd) | Beclometasone dipropionate |  |
| 3363 | Becloforte 400microgram disks with Diskhaler (GlaxoSmithKline UK Ltd) | Beclometasone dipropionate |  |
| 3546 | Qvar 50 inhaler (Teva UK Ltd) | Beclometasone dipropionate |  |
| 3743 | Filair 50 inhaler (Meda Pharmaceuticals Ltd) | Beclometasone dipropionate |  |
| 3927 | Filair 100 inhaler (Meda Pharmaceuticals Ltd) | Beclometasone dipropionate |  |
| 3947 | Becotide 100microgram Rotacaps (GlaxoSmithKline UK Ltd) | Beclometasone dipropionate |  |
| 3993 | Filair Forte 250micrograms/dose inhaler (Meda Pharmaceuticals Ltd) | Beclometasone dipropionate |  |
| 4365 | Beclometasone 100micrograms disc | Beclometasone Dipropionate |  |
| 4413 | Qvar 100 Autohaler (Teva UK Ltd) | Beclometasone dipropionate |  |
| 4499 | Aerobec 250microgram/actuation Pressurised inhalation (Meda Pharmaceuticals Ltd) | Beclometasone dipropionate |  |
| 4601 | Asmabec 100 Clickhaler (Focus Pharmaceuticals Ltd) | Beclometasone dipropionate |  |
| 4759 | Beclometasone 100microgram inhalation powder capsules | Beclometasone dipropionate |  |
| 4803 | Beclazone 250microgram/actuation Inhalation powder (Actavis UK Ltd) | Beclometasone dipropionate |  |
| 5521 | Beclometasone 200micrograms/dose dry powder inhaler | Beclometasone dipropionate |  |
| 5522 | Beclometasone 100micrograms/dose dry powder inhaler | Beclometasone dipropionate |  |
| 5804 | Beclometasone 250micrograms/dose dry powder inhaler | Beclometasone dipropionate |  |
| 5992 | Beclometasone 50micrograms/dose dry powder inhaler | Beclometasone dipropionate |  |
| 7653 | Beclometasone 400microgram inhalation powder capsules | Beclometasone dipropionate |  |
| 7964 | Beclometasone 50micrograms/ml nebuliser suspension | Beclometasone Dipropionate |  |
| 8111 | Becloforte vm 250microgram/actuation VM pack (Allen & Hanburys Ltd) | Beclometasone dipropionate |  |
| 9233 | Beclometasone 200microgram inhalation powder capsules | Beclometasone dipropionate |  |
| 9477 | Asmabec 100microgram/actuation Spacehaler (Celltech Pharma Europe Ltd) | Beclometasone Dipropionate |  |
| 9571 | Beclometasone 250micrograms/actuation vortex inhaler | Beclometasone Dipropionate |  |
| 9577 | Asmabec 50 Clickhaler (Focus Pharmaceuticals Ltd) | Beclometasone dipropionate |  |
| 9599 | Beclazone 50microgram/actuation Inhalation powder (Actavis UK Ltd) | Beclometasone dipropionate |  |
| 9921 | Beclometasone 100micrograms/dose breath actuated inhaler CFC free | Beclometasone dipropionate |  |
| 10090 | Beclometasone 50micrograms/actuation extrafine particle cfc free inhaler | Beclometasone Dipropionate |  |
| 11198 | Beclometasons 50 micrograms/actuation vortex inhaler | Beclometasone Dipropionate |  |
| 11497 | Beclometasone 400micrograms/dose dry powder inhaler | Beclometasone dipropionate |  |
| 11732 | Beclometasone 50micrograms/dose breath actuated inhaler CFC free | Beclometasone dipropionate |  |
| 13037 | Pulvinal Beclometasone Dipropionate 200micrograms/dose dry powder inhaler (Chiesi Ltd) | Beclometasone dipropionate |  |
| 13290 | Clenil Modulite 100micrograms/dose inhaler (Chiesi Ltd) | Beclometasone dipropionate |  |
| 13815 | Beclazone 100microgram/actuation Inhalation powder (Actavis UK Ltd) | Beclometasone dipropionate |  |
| 14294 | Qvar 50micrograms/dose Easi-Breathe inhaler (Teva UK Ltd) | Beclometasone dipropionate |  |
| 14321 | Beclometasone 200micrograms/dose inhaler CFC free | Beclometasone Dipropionate |  |
| 14524 | Bdp 250microgram/actuation Spacehaler (Celltech Pharma Europe Ltd) | Beclometasone Dipropionate |  |
| 14567 | Asmabec 250 Clickhaler (Focus Pharmaceuticals Ltd) | Beclometasone dipropionate |  |
| 14590 | Asmabec 250microgram/actuation Spacehaler (Celltech Pharma Europe Ltd) | Beclometasone Dipropionate |  |
| 14736 | Pulvinal Beclometasone Dipropionate 400micrograms/dose dry powder inhaler (Chiesi Ltd) | Beclometasone dipropionate |  |
| 14757 | Pulvinal Beclometasone Dipropionate 100micrograms/dose dry powder inhaler (Chiesi Ltd) | Beclometasone dipropionate |  |
| 15326 | Beclometasone 100micrograms/dose inhaler CFC free | Beclometasone dipropionate |  |
| 15706 | Beclometasone 100 micrograms/actuation vortex inhaler | Beclometasone Dipropionate |  |
| 16148 | Clenil Modulite 250micrograms/dose inhaler (Chiesi Ltd) | Beclometasone dipropionate |  |
| 16151 | Clenil Modulite 200micrograms/dose inhaler (Chiesi Ltd) | Beclometasone Dipropionate |  |
| 16158 | Clenil Modulite 50micrograms/dose inhaler (Chiesi Ltd) | Beclometasone dipropionate |  |
| 16584 | Beclometasone 50micrograms/dose inhaler CFC free | Beclometasone dipropionate |  |
| 17654 | Easyhaler Beclometasone 200micrograms/dose dry powder inhaler (Orion Pharma (UK) Ltd) | Beclometasone dipropionate |  |
| 18394 | Bdp 50microgram/actuation Spacehaler (Celltech Pharma Europe Ltd) | Beclometasone Dipropionate |  |
| 18848 | Qvar 100micrograms/dose Easi-Breathe inhaler (Teva UK Ltd) | Beclometasone dipropionate |  |
| 19031 | Bdp 100microgram/actuation Spacehaler (Celltech Pharma Europe Ltd) | Beclometasone Dipropionate |  |
| 19389 | Asmabec 50microgram/actuation Spacehaler (Celltech Pharma Europe Ltd) | Beclometasone Dipropionate |  |
| 19401 | Beclometasone 250micrograms/actuation inhaler and compact spacer | Beclometasone Dipropionate |  |
| 20825 | Spacehaler BDP 250microgram/actuation Spacehaler (Celltech Pharma Europe Ltd) | Beclometasone Dipropionate |  |
| 21005 | Beclometasone 250micrograms/dose inhaler CFC free | Beclometasone dipropionate |  |
| 21482 | Beclometasone 100micrograms/dose inhaler (Mylan Ltd) | Beclometasone dipropionate |  |
| 24898 | Spacehaler BDP 100microgram/actuation Spacehaler (Celltech Pharma Europe Ltd) | Beclometasone Dipropionate |  |
| 25204 | Beclometasone 100micrograms/dose inhaler (A A H Pharmaceuticals Ltd) | Beclometasone dipropionate |  |
| 26063 | Beclometasone 100micrograms/dose inhaler (Teva UK Ltd) | Beclometasone dipropionate |  |
| 27679 | Beclometasone 100microgram/actuation Pressurised inhalation (Approved Prescription Services Ltd) | Beclometasone dipropionate |  |
| 28073 | Beclometasone 250microgram/actuation Pressurised inhalation (Approved Prescription Services Ltd) | Beclometasone dipropionate |  |
| 28640 | Beclometasone 100microgram/actuation Inhalation powder (Actavis UK Ltd) | Beclometasone dipropionate |  |
| 28761 | Spacehaler BDP 50microgram/actuation Spacehaler (Celltech Pharma Europe Ltd) | Beclometasone Dipropionate |  |
| 29325 | Beclometasone 250micrograms/dose inhaler (Mylan Ltd) | Beclometasone dipropionate |  |
| 30210 | Beclometasone 250micrograms/dose inhaler (Teva UK Ltd) | Beclometasone dipropionate |  |
| 30238 | Beclometasone 50microgram/actuation Pressurised inhalation (Approved Prescription Services Ltd) | Beclometasone dipropionate |  |
| 31774 | Beclometasone 50micrograms/dose inhaler (Mylan Ltd) | Beclometasone dipropionate |  |
| 32874 | Beclometasone 50microgram/actuation Inhalation powder (Actavis UK Ltd) | Beclometasone dipropionate |  |
| 33258 | Beclometasone 250micrograms/dose inhaler (A A H Pharmaceuticals Ltd) | Beclometasone dipropionate |  |
| 33849 | Beclometasone 100microgram/actuation Inhalation powder (Neo Laboratories Ltd) | Beclometasone dipropionate |  |
| 34315 | Beclometasone 250microgram/actuation Inhalation powder (Actavis UK Ltd) | Beclometasone dipropionate |  |
| 34428 | Beclometasone 50microgram/actuation Inhalation powder (Neo Laboratories Ltd) | Beclometasone dipropionate |  |
| 34739 | Beclometasone 50micrograms/dose inhaler (Teva UK Ltd) | Beclometasone dipropionate |  |
| 34794 | Beclometasone 200micrograms/dose inhaler (A A H Pharmaceuticals Ltd) | Beclometasone dipropionate |  |
| 34859 | Beclometasone 250microgram/actuation Inhalation powder (Neo Laboratories Ltd) | Beclometasone dipropionate |  |
| 34919 | Beclometasone 50micrograms/dose inhaler (A A H Pharmaceuticals Ltd) | Beclometasone dipropionate |  |
| 41269 | Beclometasone 400 Cyclocaps (Teva UK Ltd) | Beclometasone dipropionate |  |
| 41412 | Beclometasone 400micrograms/actuation inhaler | Beclometasone Dipropionate |  |
| 46157 | Beclometasone 200 Cyclocaps (Teva UK Ltd) | Beclometasone dipropionate |  |
| 3556 | Beclometasone 50micrograms with salbutamol 100micrograms/inhalation inhaler | Beclometasone Dipropionate/Salbutamol |  |
| 19121 | Beclometasone 100micrograms with Salbutamol 200micrograms inhalation capsules | Beclometasone Dipropionate/Salbutamol |  |
| 19376 | Beclometasone 200micrograms with Salbutamol 400micrograms inhalation capsules | Beclometasone Dipropionate/Salbutamol |  |
| 14561 | Salbutamol 400microgram / Beclometasone 200microgram inhalation powder capsules | Beclometasone dipropionate/Salbutamol sulfate |  |
| 16625 | Ventide Rotacaps (GlaxoSmithKline UK Ltd) | Beclometasone dipropionate/Salbutamol sulfate |  |
| 18456 | Salbutamol 200microgram / Beclometasone 100microgram inhalation powder capsules | Beclometasone dipropionate/Salbutamol sulfate |  |
| 18484 | Ventide Paediatric Rotacaps (GlaxoSmithKline UK Ltd) | Beclometasone dipropionate/Salbutamol sulfate |  |
| 454 | Pulmicort 200microgram Inhaler (AstraZeneca UK Ltd) | Budesonide |  |
| 908 | Pulmicort 400 Turbohaler (AstraZeneca UK Ltd) | Budesonide |  |
| 909 | Budesonide 200micrograms/dose inhaler | Budesonide |  |
| 947 | Budesonide 50micrograms/actuation refill canister | Budesonide |  |
| 956 | Pulmicort 200 Turbohaler (AstraZeneca UK Ltd) | Budesonide |  |
| 959 | Budesonide 50micrograms/dose inhaler | Budesonide |  |
| 960 | Pulmicort 100 Turbohaler (AstraZeneca UK Ltd) | Budesonide |  |
| 1642 | Budesonide 400micrograms/dose dry powder inhaler | Budesonide |  |
| 1680 | Pulmicort LS 50micrograms/dose inhaler (AstraZeneca UK Ltd) | Budesonide |  |
| 1956 | Pulmicort 1mg Respules (AstraZeneca UK Ltd) | Budesonide |  |
| 1959 | Pulmicort 0.5mg Respules (AstraZeneca UK Ltd) | Budesonide |  |
| 2092 | Budesonide 200micrograms/dose dry powder inhaler | Budesonide |  |
| 2125 | Pulmicort 200microgram Refill canister (AstraZeneca UK Ltd) | Budesonide |  |
| 3570 | Budesonide 200micrograms/actuation refill canister | Budesonide |  |
| 4545 | Pulmicort LS 50microgram Refill canister (AstraZeneca UK Ltd) | Budesonide |  |
| 4801 | Budesonide 500micrograms/2ml nebuliser liquid unit dose vials | Budesonide |  |
| 4942 | Budesonide 1mg/2ml nebuliser liquid unit dose vials | Budesonide |  |
| 7788 | Budesonide 100micrograms/dose dry powder inhaler | Budesonide |  |
| 8433 | Budesonide 100micrograms/actuation inhaler | Budesonide |  |
| 10321 | Budesonide 400microgram inhalation powder capsules | Budesonide |  |
| 14700 | Budesonide 400micrograms/actuation inhaler | Budesonide |  |
| 16054 | Budesonide 200micrograms/actuation breath actuated powder inhaler | Budesonide |  |
| 17670 | Easyhaler Budesonide 100micrograms/dose dry powder inhaler (Orion Pharma (UK) Ltd) | Budesonide |  |
| 18537 | Budesonide 200microgram inhalation powder capsules | Budesonide |  |
| 23741 | Novolizer budesonide 200microgram/actuation Pressurised inhalation (Meda Pharmaceuticals Ltd) | Budesonide |  |
| 27188 | Easyhaler Budesonide 200micrograms/dose dry powder inhaler (Orion Pharma (UK) Ltd) | Budesonide |  |
| 30649 | Easyhaler Budesonide 400micrograms/dose dry powder inhaler (Orion Pharma (UK) Ltd) | Budesonide |  |
| 35510 | Budesonide 200micrograms/dose dry powder inhalation cartridge with device | Budesonide |  |
| 35602 | Budesonide 200micrograms/dose dry powder inhalation cartridge | Budesonide |  |
| 35631 | Budelin Novolizer 200micrograms/dose inhalation powder (Meda Pharmaceuticals Ltd) | Budesonide |  |
| 35724 | Budelin Novolizer 200micrograms/dose inhalation powder refill (Meda Pharmaceuticals Ltd) | Budesonide |  |
| 6325 | Symbicort 200/6 Turbohaler (AstraZeneca UK Ltd) | Budesonide/Formoterol fumarate dihydrate |  |
| 6746 | Budesonide 400micrograms/dose / Formoterol 12micrograms/dose dry powder inhaler | Budesonide/Formoterol fumarate dihydrate |  |
| 6780 | Symbicort 400/12 Turbohaler (AstraZeneca UK Ltd) | Budesonide/Formoterol fumarate dihydrate |  |
| 6796 | Budesonide 200micrograms/dose / Formoterol 6micrograms/dose dry powder inhaler | Budesonide/Formoterol fumarate dihydrate |  |
| 7013 | Symbicort 100/6 Turbohaler (AstraZeneca UK Ltd) | Budesonide/Formoterol fumarate dihydrate |  |
| 10218 | Budesonide 100micrograms/dose / Formoterol 6micrograms/dose dry powder inhaler | Budesonide/Formoterol fumarate dihydrate |  |
| 3187 | Choledyl 62.5mg/5ml Oral solution (Parke-davis Research Laboratories) | Choline Hydrogen Tartrate |  |
| 4591 | Choledyl 100mg Tablet (Parke-davis Research Laboratories) | Choline Hydrogen Tartrate | 1 |
| 4592 | Choledyl 200mg Tablet (Parke-davis Research Laboratories) | Choline Hydrogen Tartrate | 1 |
| 7832 | Choline theophyllinate 200mg tablets | Choline Hydrogen Tartrate | 1 |
| 18288 | Choline theophyllinate 100mg tablets | Choline Hydrogen Tartrate | 1 |
| 18988 | Choline theophyllinate 62.5mg/5ml oral solution | Choline Hydrogen Tartrate |  |
| 6839 | Alvesco 160 inhaler (Takeda UK Ltd) | Ciclesonide |  |
| 7356 | Ciclesonide 80micrograms/dose inhaler CFC free | Ciclesonide |  |
| 10102 | Ciclesonide 160micrograms/dose inhaler CFC free | Ciclesonide |  |
| 21224 | Alvesco 80 inhaler (Takeda UK Ltd) | Ciclesonide |  |
| 1794 | Berotec 100microgram/actuation Inhalation powder (Boehringer Ingelheim Ltd) | Fenoterol Hydrobromide |  |
| 2020 | Berotec 200micrograms/dose inhaler (Boehringer Ingelheim Ltd) | Fenoterol hydrobromide |  |
| 4842 | Fenoterol 100microgram/actuation inhaler | Fenoterol Hydrobromide |  |
| 5185 | Fenoterol 200micrograms/dose inhaler | Fenoterol hydrobromide |  |
| 13365 | Berotec 5mg/ml Nebuliser liquid (Boehringer Ingelheim Ltd) | Fenoterol Hydrobromide |  |
| 2722 | Duovent inhaler (Boehringer Ingelheim Ltd) | Fenoterol hydrobromide/Ipratropium bromide |  |
| 3786 | Fenoterol 100micrograms/dose / Ipratropium 40micrograms/dose inhaler | Fenoterol hydrobromide/Ipratropium bromide |  |
| 16207 | Duovent UDVs nebuliser liquid 4ml (Boehringer Ingelheim Ltd) | Fenoterol hydrobromide/Ipratropium bromide |  |
| 18299 | Fenoterol 1.25mg/4ml / Ipratropium 500micrograms/4ml nebuliser liquid unit dose vials | Fenoterol hydrobromide/Ipratropium bromide |  |
| 911 | Flixotide accuhaler 250 250microgram/inhalation Inhalation powder (Allen & Hanburys Ltd) | Fluticasone propionate |  |
| 1412 | Flixotide 250microgram/actuation Inhalation powder (Allen & Hanburys Ltd) | Fluticasone Propionate |  |
| 1424 | Flixotide 250microgram Disc (Allen & Hanburys Ltd) | Fluticasone Propionate |  |
| 1426 | Flixotide 500microgram Disc (Allen & Hanburys Ltd) | Fluticasone Propionate |  |
| 1518 | Flixotide 50microgram/actuation Inhalation powder (Allen & Hanburys Ltd) | Fluticasone Propionate |  |
| 1676 | Flixotide 125microgram/actuation Inhalation powder (Allen & Hanburys Ltd) | Fluticasone Propionate |  |
| 2282 | Fluticasone propionate 500micrograms/dose dry powder inhaler | Fluticasone propionate |  |
| 2440 | Flixotide accuhaler 500 500microgram/inhalation Inhalation powder (Allen & Hanburys Ltd) | Fluticasone propionate |  |
| 2723 | Fluticasone 25micrograms/dose inhaler | Fluticasone propionate |  |
| 2951 | Fluticasone 250microgram/actuation Pressurised inhalation | Fluticasone Propionate |  |
| 3289 | Flixotide 25micrograms/dose inhaler (GlaxoSmithKline UK Ltd) | Fluticasone propionate |  |
| 3989 | Flixotide 100microgram Disc (Allen & Hanburys Ltd) | Fluticasone Propionate |  |
| 4131 | Fluticasone 100microgram Disc | Fluticasone Propionate |  |
| 4132 | Fluticasone 125microgram/actuation Pressurised inhalation | Fluticasone Propionate |  |
| 4688 | Fluticasone 50microgram/actuation Pressurised inhalation | Fluticasone Propionate |  |
| 4926 | Flixotide accuhaler 100 100microgram/inhalation Inhalation powder (Allen & Hanburys Ltd) | Fluticasone propionate |  |
| 5223 | Fluticasone 50micrograms/dose inhaler CFC free | Fluticasone propionate |  |
| 5309 | Flixotide 50micrograms/dose Evohaler (GlaxoSmithKline UK Ltd) | Fluticasone propionate |  |
| 5551 | Flixotide 0.5mg/2ml Nebules (GlaxoSmithKline UK Ltd) | Fluticasone propionate |  |
| 5580 | Flixotide accuhaler 50 50microgram/inhalation Inhalation powder (Allen & Hanburys Ltd) | Fluticasone propionate |  |
| 5683 | Flixotide 250micrograms/dose Evohaler (GlaxoSmithKline UK Ltd) | Fluticasone propionate |  |
| 5718 | Flixotide 125micrograms/dose Evohaler (GlaxoSmithKline UK Ltd) | Fluticasone propionate |  |
| 5822 | Fluticasone 250micrograms/dose inhaler CFC free | Fluticasone propionate |  |
| 5885 | Fluticasone propionate 100micrograms/dose dry powder inhaler | Fluticasone propionate |  |
| 5975 | Fluticasone 125micrograms/dose inhaler CFC free | Fluticasone propionate |  |
| 7602 | Fluticasone 50microgram Disc | Fluticasone Propionate |  |
| 7638 | Fluticasone 250microgram Disc | Fluticasone Propionate |  |
| 7891 | Fluticasone 500microgram Disc | Fluticasone Propionate |  |
| 7948 | Fluticasone propionate 250micrograms/dose dry powder inhaler | Fluticasone propionate |  |
| 8635 | Flixotide 50microgram Disc (Allen & Hanburys Ltd) | Fluticasone Propionate |  |
| 9164 | Fluticasone propionate 50micrograms/dose dry powder inhaler | Fluticasone propionate |  |
| 11478 | Fluticasone 2mg/2ml nebuliser liquid unit dose vials | Fluticasone propionate |  |
| 16305 | Flixotide 2mg/2ml Nebules (GlaxoSmithKline UK Ltd) | Fluticasone propionate |  |
| 17465 | Fluticasone 500micrograms/2ml nebuliser liquid unit dose vials | Fluticasone propionate |  |
| 638 | Seretide 250 Accuhaler (GlaxoSmithKline UK Ltd) | Fluticasone propionate/Salmeterol xinafoate |  |
| 5143 | Seretide 50 Evohaler (GlaxoSmithKline UK Ltd) | Fluticasone propionate/Salmeterol xinafoate |  |
| 5161 | Seretide 125 Evohaler (GlaxoSmithKline UK Ltd) | Fluticasone propionate/Salmeterol xinafoate |  |
| 5172 | Seretide 250 Evohaler (GlaxoSmithKline UK Ltd) | Fluticasone propionate/Salmeterol xinafoate |  |
| 11588 | Fluticasone 125micrograms/dose / Salmeterol 25micrograms/dose inhaler CFC free | Fluticasone propionate/Salmeterol xinafoate |  |
| 11618 | Fluticasone 250micrograms/dose / Salmeterol 25micrograms/dose inhaler CFC free | Fluticasone propionate/Salmeterol xinafoate |  |
| 12994 | Fluticasone 50micrograms/dose / Salmeterol 25micrograms/dose inhaler CFC free | Fluticasone propionate/Salmeterol xinafoate |  |
| 13040 | Fluticasone propionate 250micrograms/dose / Salmeterol 50micrograms/dose dry powder inhaler | Fluticasone propionate/Salmeterol xinafoate |  |
| 1974 | Oxis 12 Turbohaler (AstraZeneca UK Ltd) | Formoterol fumarate dihydrate |  |
| 1975 | Oxis 6 Turbohaler (AstraZeneca UK Ltd) | Formoterol fumarate dihydrate |  |
| 6526 | Formoterol 12microgram inhalation powder capsules with device | Formoterol fumarate dihydrate |  |
| 7133 | Formoterol 12micrograms/dose dry powder inhaler | Formoterol fumarate dihydrate |  |
| 9711 | Formoterol 6micrograms/dose dry powder inhaler | Formoterol fumarate dihydrate |  |
| 10968 | Foradil 12microgram inhalation powder capsules with device (Novartis Pharmaceuticals UK Ltd) | Formoterol fumarate dihydrate |  |
| 14306 | Formoterol 12micrograms/dose inhaler CFC free | Formoterol Fumarate Dihydrate |  |
| 25784 | Atimos Modulite 12micrograms/dose inhaler (Chiesi Ltd) | Formoterol fumarate dihydrate |  |
| 534 | Atrovent 20micrograms/dose inhaler (Boehringer Ingelheim Ltd) | Ipratropium bromide |  |
| 1409 | Ipratropium bromide 20micrograms/dose inhaler | Ipratropium bromide |  |
| 1410 | Ipratropium bromide 0.25mg/ml | Ipratropium Bromide |  |
| 1411 | Ipratropium bromide 250micrograms/ml | Ipratropium Bromide |  |
| 1415 | Steri-neb ipratropium 250microgram/ml Nebuliser liquid (IVAX Pharmaceuticals UK Ltd) | Ipratropium Bromide |  |
| 1697 | Atrovent 20micrograms/dose Autohaler (Boehringer Ingelheim Ltd) | Ipratropium bromide |  |
| 1962 | Atrovent udv 0.25mg/ml Nebuliser liquid (Boehringer Ingelheim Ltd) | Ipratropium Bromide |  |
| 2994 | Atrovent aerocaps 40microgram Inhalation powder (Boehringer Ingelheim Ltd) | Ipratropium bromide |  |
| 3306 | Atrovent Forte 40micrograms/dose inhaler (Boehringer Ingelheim Ltd) | Ipratropium bromide |  |
| 4268 | Ipratropium bromide 40micrograms/dose inhaler | Ipratropium bromide |  |
| 6081 | Ipratropium bromide 20micrograms/dose breath actuated inhaler | Ipratropium bromide |  |
| 6512 | Atrovent 20micrograms/dose inhaler CFC free (Boehringer Ingelheim Ltd) | Ipratropium bromide |  |
| 6522 | Ipratropium bromide 20micrograms/dose inhaler CFC free | Ipratropium bromide |  |
| 6719 | Ipratropium bromide 500micrograms/2ml nebuliser liquid unit dose vials | Ipratropium bromide |  |
| 6758 | Ipratropium 250micrograms/1ml nebuliser liquid Steri-Neb unit dose vials (Teva UK Ltd) | Ipratropium bromide |  |
| 6772 | Ipratropium bromide 250micrograms/1ml nebuliser liquid unit dose vials | Ipratropium bromide |  |
| 6911 | Atrovent 250micrograms/1ml nebuliser liquid UDVs (Boehringer Ingelheim Ltd) | Ipratropium bromide |  |
| 7140 | Atrovent 500micrograms/2ml nebuliser liquid UDVs (Boehringer Ingelheim Ltd) | Ipratropium bromide |  |
| 8333 | Ipratropium bromide 40microgram inhalation powder capsules | Ipratropium bromide |  |
| 9681 | Atrovent aerohaler 40microgram Inhalation powder (Boehringer Ingelheim Ltd) | Ipratropium bromide |  |
| 11779 | Ipratropium bromide 40microgram inhalation powder capsules with device | Ipratropium bromide |  |
| 13757 | Tropiovent steripoule 250microgram/ml Nebuliser liquid (Ashbourne Pharmaceuticals Ltd) | Ipratropium Bromide |  |
| 18140 | Respontin 500micrograms/2ml Nebules (GlaxoSmithKline UK Ltd) | Ipratropium bromide |  |
| 18421 | Respontin nebules 250microgram/ml Nebuliser liquid (Glaxo Wellcome UK Ltd) | Ipratropium Bromide |  |
| 23567 | Respontin 250micrograms/1ml Nebules (GlaxoSmithKline UK Ltd) | Ipratropium bromide |  |
| 23709 | Ipratropium 500micrograms/2ml nebuliser liquid Steri-Neb unit dose vials (Teva UK Ltd) | Ipratropium bromide |  |
| 23961 | Ipratropium bromide 250microgram/ml Inhalation vapour (Galen Ltd) | Ipratropium Bromide |  |
| 30229 | Ipratropium bromide 250microgram/ml Nebuliser liquid (Galen Ltd) | Ipratropium bromide |  |
| 37791 | Ipratropium bromide 250microgram/ml | Ipratropium Bromide |  |
| 40177 | Ipratropium bromide 250microgram/ml Nebuliser liquid (Hillcross Pharmaceuticals Ltd) | Ipratropium Bromide |  |
| 2862 | Duovent Autohaler (Boehringer Ingelheim Ltd) | Ipratropium bromide/Fenoterol hydrobromide |  |
| 9270 | Ipratropium bromide with fenoterol hydrobromide 500micrograms + 1.25mg/4ml | Ipratropium Bromide/Fenoterol Hydrobromide |  |
| 12808 | Fenoterol 100micrograms/dose / Ipratropium bromide 40micrograms/dose breath actuated inhaler | Ipratropium bromide/Fenoterol hydrobromide |  |
| 26616 | Ipratropium bromide with fenoterol hydrobromide 0micrograms + 100micrograms/actuation | Ipratropium Bromide/Fenoterol Hydrobromide |  |
| 27505 | Ipratropium bromide with fenoterol hydrobromide 40micrograms + 100micrograms/actuation | Ipratropium Bromide/Fenoterol Hydrobromide |  |
| 3305 | Combivent nebuliser liquid 2.5ml UDVs (Boehringer Ingelheim Ltd) | Ipratropium bromide/Salbutamol sulfate |  |
| 35557 | Ipramol nebuliser solution 2.5ml Steri-Neb unit dose vials (Teva UK Ltd) | Ipratropium bromide/Salbutamol sulfate |  |
| 3374 | Ketotifen 1mg tablets | Ketotifen fumarate | 1 |
| 3787 | Zaditen 1mg Tablet (Novartis Pharmaceuticals UK Ltd) | Ketotifen fumarate | 1 |
| 9635 | Zaditen 1mg/5ml Oral solution (Novartis Pharmaceuticals UK Ltd) | Ketotifen fumarate |  |
| 10812 | Zaditen 1mg capsules (Novartis Pharmaceuticals UK Ltd) | Ketotifen fumarate |  |
| 10813 | Ketotifen 1mg capsules | Ketotifen fumarate |  |
| 10254 | Mometasone 400micrograms/dose dry powder inhaler | Mometasone furoate |  |
| 16018 | Mometasone 200micrograms/dose dry powder inhaler | Mometasone furoate |  |
| 16433 | Asmanex 200micrograms/dose Twisthaler (Merck Sharp & Dohme Ltd) | Mometasone furoate |  |
| 17590 | Asmanex 400micrograms/dose Twisthaler (Merck Sharp & Dohme Ltd) | Mometasone furoate |  |
| 622 | Montelukast 4mg chewable tablets sugar free | Montelukast sodium | 1 |
| 695 | Singulair 10mg tablets (Merck Sharp & Dohme Ltd) | Montelukast sodium | 1 |
| 808 | Montelukast 10mg tablets | Montelukast sodium | 1 |
| 5594 | Singulair Paediatric 5mg chewable tablets (Merck Sharp & Dohme Ltd) | Montelukast sodium | 1 |
| 5957 | Montelukast 5mg chewable tablets sugar free | Montelukast sodium | 1 |
| 7088 | Montelukast 4mg granules sachets sugar free | Montelukast sodium |  |
| 14162 | Singulair Paediatric 4mg chewable tablets (Merck Sharp & Dohme Ltd) | Montelukast sodium | 1 |
| 14200 | Singulair Paediatric 4mg granules sachets (Merck Sharp & Dohme Ltd) | Montelukast sodium |  |
| 48396 | Singulair 10mg tablets (Necessity Supplies Ltd) | Montelukast sodium | 1 |
| 56604 | Montelukast 4mg chewable tablets sugar free (Actavis UK Ltd) | Montelukast sodium | 1 |
| 56756 | Montelukast 4mg granules sachets sugar free (A A H Pharmaceuticals Ltd) | Montelukast sodium |  |
| 57621 | Singulair Paediatric 4mg granules sachets (Mawdsley-Brooks & Company Ltd) | Montelukast sodium |  |
| 59263 | Montelukast 10mg tablets (Teva UK Ltd) | Montelukast sodium | 1 |
| 59819 | Montelukast 10mg tablets (Actavis UK Ltd) | Montelukast sodium | 1 |
| 59968 | Montelukast 5mg chewable tablets sugar free (Teva UK Ltd) | Montelukast sodium | 1 |
| 60331 | Montelukast 10mg tablets (Ranbaxy (UK) Ltd) | Montelukast sodium | 1 |
| 62410 | Montelukast 10mg tablets (Alliance Healthcare (Distribution) Ltd) | Montelukast sodium | 1 |
| 62490 | Montelukast 10mg tablets (A A H Pharmaceuticals Ltd) | Montelukast sodium | 1 |
| 63457 | Montelukast 5mg chewable tablets sugar free (Accord Healthcare Ltd) | Montelukast sodium | 1 |
| 64444 | Singulair 10mg tablets (DE Pharmaceuticals) | Montelukast sodium | 1 |
| 64648 | Montelukast 10mg tablets (Milpharm Ltd) | Montelukast sodium | 1 |
| 65038 | Montelukast 10mg tablets (Accord Healthcare Ltd) | Montelukast sodium | 1 |
| 3688 | Tilade 2mg/dose inhaler (Sanofi) | Nedocromil sodium |  |
| 8215 | Tilade 2mg/inhalation Inhalation powder (Sanofi) | Nedocromil Sodium |  |
| 8608 | Nedocromil sodium 2mg/inhalation inhaler | Nedocromil Sodium |  |
| 10597 | Tilade 2mg/dose Syncroner with spacer (Sanofi) | Nedocromil sodium |  |
| 12633 | Tilarin 1% Nasal spray suspension (Rhone-Poulenc Rorer Ltd) | Nedocromil Sodium |  |
| 13256 | Nedocromil 2mg/dose inhaler CFC free | Nedocromil Sodium |  |
| 14448 | Nedocromil sodium 1% nasal spray | Nedocromil Sodium |  |
| 25119 | Tilade 2mg/dose inhaler CFC free (Sanofi) | Nedocromil Sodium |  |
| 8 | Salbutamol 100micrograms/dose inhaler | Salbutamol |  |
| 31 | Ventolin 100microgram/inhalation Inhalation powder (Glaxo Wellcome UK Ltd) | Salbutamol |  |
| 862 | Salbulin Inhalation powder (3M Health Care Ltd) | Salbutamol |  |
| 1093 | Salamol 100microgram/actuation Inhalation powder (IVAX Pharmaceuticals UK Ltd) | Salbutamol |  |
| 2978 | Salbutamol 200micrograms/dose dry powder inhaler | Salbutamol |  |
| 3443 | Salbutamol 100microgram/inhalation Spacehaler (Celltech Pharma Europe Ltd) | Salbutamol |  |
| 4497 | Ventolin accuhaler 200 200microgram/actuation Inhalation powder (Glaxo Wellcome UK Ltd) | Salbutamol |  |
| 7017 | Salbutamol 100micrograms/dose dry powder inhaler | Salbutamol |  |
| 7935 | Maxivent 100microgram/inhalation Inhalation powder (Ashbourne Pharmaceuticals Ltd) | Salbutamol |  |
| 9651 | Asmasal 100microgram/inhalation Spacehaler (Celltech Pharma Europe Ltd) | Salbutamol |  |
| 13038 | Pulvinal Salbutamol 200micrograms/dose dry powder inhaler (Chiesi Ltd) | Salbutamol |  |
| 13181 | Easyhaler Salbutamol sulfate 100micrograms/dose dry powder inhaler (Orion Pharma (UK) Ltd) | Salbutamol |  |
| 14525 | Salbutamol 100micrograms/inhalation vortex inhaler | Salbutamol |  |
| 16577 | Easyhaler Salbutamol sulfate 200micrograms/dose dry powder inhaler (Orion Pharma (UK) Ltd) | Salbutamol |  |
| 21859 | Asmaven 100microgram Inhalation powder (Berk Pharmaceuticals Ltd) | Salbutamol |  |
| 22430 | Spacehaler salbutamol 100microgram/inhalation Spacehaler (Celltech Pharma Europe Ltd) | Salbutamol |  |
| 28508 | Salbutamol 100microgram/inhalation Inhalation powder (IVAX Pharmaceuticals UK Ltd) | Salbutamol |  |
| 31933 | Salbutamol 100micrograms/dose inhaler (A A H Pharmaceuticals Ltd) | Salbutamol |  |
| 33089 | Salbutamol 100micrograms/dose inhaler (Kent Pharmaceuticals Ltd) | Salbutamol |  |
| 33588 | Salbutamol 100micrograms/dose inhaler (Mylan Ltd) | Salbutamol |  |
| 34134 | Aerolin 400 100microgram/actuation Inhalation powder (3M Health Care Ltd) | Salbutamol |  |
| 34311 | Salbutamol 100microgram/inhalation Inhalation powder (Berk Pharmaceuticals Ltd) | Salbutamol |  |
| 34702 | Salbutamol 100microgram/inhalation Inhalation powder (C P Pharmaceuticals Ltd) | Salbutamol |  |
| 40655 | Salbuvent 100microgram/actuation Inhalation powder (Pharmacia Ltd) | Salbutamol |  |
| 42497 | Salbutamol 8mg tablet | Salbutamol | 1 |
| 44713 | Salbutamol 100microgram/inhalation Inhalation powder (Celltech Pharma Europe Ltd) | Salbutamol |  |
| 17 | Salbutamol 100micrograms/dose inhaler CFC free | Salbutamol sulfate |  |
| 282 | Salbutamol 2mg/5ml oral solution sugar free | Salbutamol sulfate |  |
| 510 | Ventolin 5mg/ml respirator solution (GlaxoSmithKline UK Ltd) | Salbutamol sulfate |  |
| 674 | Ventolin 2.5mg Nebules (GlaxoSmithKline UK Ltd) | Salbutamol sulfate |  |
| 696 | Salbutamol 8mg modified-release capsules | Salbutamol sulfate |  |
| 856 | Ventolin 2mg/5ml syrup (GlaxoSmithKline UK Ltd) | Salbutamol sulfate |  |
| 881 | Salbutamol 2mg tablets | Salbutamol sulfate | 1 |
| 882 | Salbutamol 200microgram inhalation powder capsules | Salbutamol sulfate |  |
| 898 | Ventolin evohaler 100 100microgram/inhalation Pressurised inhalation (Glaxo Wellcome UK Ltd) | Salbutamol sulfate |  |
| 942 | Aerolin 100micrograms/dose Autohaler (3M Health Care Ltd) | Salbutamol sulfate |  |
| 957 | Salamol easi-breathe 100microgram/actuation Pressurised inhalation (IVAX Pharmaceuticals UK Ltd) | Salbutamol sulfate |  |
| 958 | Ventolin easi-breathe 100microgram/actuation Pressurised inhalation (Allen & Hanburys Ltd) | Salbutamol sulfate |  |
| 987 | Ventolin 4mg Tablet (Allen & Hanburys Ltd) | Salbutamol sulfate | 1 |
| 1087 | Asmasal 95micrograms/dose Clickhaler (Focus Pharmaceuticals Ltd) | Salbutamol sulfate |  |
| 1414 | Salamol 5mg/2.5ml nebuliser liquid Steri-Neb unit dose vials (Teva UK Ltd) | Salbutamol sulfate |  |
| 1630 | Salbutamol 2.5mg/2.5ml nebuliser liquid unit dose vials | Salbutamol sulfate |  |
| 1635 | Salbuvent 2mg/5ml Oral solution (Pharmacia Ltd) | Salbutamol sulfate |  |
| 1698 | Salbutamol 100micrograms/dose breath actuated inhaler | Salbutamol sulfate |  |
| 1711 | Salbutamol 5mg/2.5ml nebuliser liquid unit dose vials | Salbutamol sulfate |  |
| 1741 | Salbutamol 100micrograms/dose breath actuated inhaler CFC free | Salbutamol sulfate |  |
| 1952 | Ventolin 400microgram Rotacaps (GlaxoSmithKline UK Ltd) | Salbutamol sulfate |  |
| 1957 | Ventolin 5mg Nebules (GlaxoSmithKline UK Ltd) | Salbutamol sulfate |  |
| 1960 | Volmax 8mg modified-release tablets (GlaxoSmithKline UK Ltd) | Salbutamol sulfate | 1 |
| 1961 | Volmax 4mg modified-release tablets (GlaxoSmithKline UK Ltd) | Salbutamol sulfate | 1 |
| 2655 | Airomir 100micrograms/dose inhaler (Teva UK Ltd) | Salbutamol sulfate |  |
| 2850 | Salbutamol 400microgram inhalation powder capsules | Salbutamol sulfate |  |
| 2851 | Ventolin 200microgram Rotacaps (GlaxoSmithKline UK Ltd) | Salbutamol sulfate |  |
| 2869 | Salbutamol 8mg modified-release tablets | Salbutamol sulfate | 1 |
| 3254 | Salbulin 4mg Tablet (3M Health Care Ltd) | Salbutamol sulfate | 1 |
| 3994 | Salbutamol 4mg modified-release tablets | Salbutamol sulfate | 1 |
| 4055 | Salbulin 2mg/5ml Oral solution (3M Health Care Ltd) | Salbutamol sulfate |  |
| 4171 | Ventolin 2mg Tablet (Allen & Hanburys Ltd) | Salbutamol sulfate | 1 |
| 4634 | Salamol 2.5mg/2.5ml nebuliser liquid Steri-Neb unit dose vials (Teva UK Ltd) | Salbutamol sulfate |  |
| 4665 | Salbulin 100micrograms/dose inhaler (3M Health Care Ltd) | Salbutamol sulfate |  |
| 5170 | Salamol 100micrograms/dose inhaler CFC free (Teva UK Ltd) | Salbutamol sulfate |  |
| 5516 | Salamol 100micrograms/dose Easi-Breathe inhaler (Teva UK Ltd) | Salbutamol sulfate |  |
| 5740 | Airomir 100micrograms/dose Autohaler (Teva UK Ltd) | Salbutamol sulfate |  |
| 5837 | Salamol steri-neb 5mg/2.5ml Nebuliser liquid (Numark Management Ltd) | Salbutamol sulfate |  |
| 5889 | Salamol 100microgram/inhalation Inhalation powder (Kent Pharmaceuticals Ltd) | Salbutamol sulfate |  |
| 5898 | Salamol steri-neb 2.5mg/2.5ml Nebuliser liquid (Numark Management Ltd) | Salbutamol sulfate |  |
| 6462 | Salbutamol 95micrograms/dose dry powder inhaler | Salbutamol sulfate |  |
| 7965 | Salbutamol 5mg/ml nebuliser liquid | Salbutamol sulfate |  |
| 9384 | Salbutamol 4mg modified-release capsules | Salbutamol sulfate |  |
| 10458 | Ventolin cr 4mg Tablet (Allen & Hanburys Ltd) | Salbutamol sulfate | 1 |
| 12042 | Ventolin cr 8mg Tablet (Allen & Hanburys Ltd) | Salbutamol sulfate | 1 |
| 13996 | Salamol 100microgram/inhalation Inhalation powder (Sandoz Ltd) | Salbutamol sulfate |  |
| 15613 | Salbutamol 500micrograms/1ml solution for injection ampoules | Salbutamol sulfate |  |
| 17185 | Ventolin 500micrograms/1ml solution for injection ampoules (GlaxoSmithKline UK Ltd) | Salbutamol sulfate |  |
| 17696 | Ventmax SR 4mg capsules (Chiesi Ltd) | Salbutamol sulfate |  |
| 18622 | Salbulin 2mg Tablet (3M Health Care Ltd) | Salbutamol sulfate | 1 |
| 18968 | Salbutamol 5mg/5ml solution for infusion ampoules | Salbutamol sulfate |  |
| 20838 | Salbuvent 2mg Tablet (Pharmacia Ltd) | Salbutamol sulfate | 1 |
| 21102 | Salbutamol 2mg/5ml Oral solution (Lagap) | Salbutamol sulfate |  |
| 22313 | Ventmax SR 8mg capsules (Chiesi Ltd) | Salbutamol sulfate |  |
| 23269 | Maxivent 2.5mg/2.5ml nebuliser liquid unit dose Steripoule vials (Ashbourne Pharmaceuticals Ltd) | Salbutamol sulfate |  |
| 25339 | Maxivent 5mg/2.5ml nebuliser liquid unit dose Steripoule vials (Ashbourne Pharmaceuticals Ltd) | Salbutamol sulfate |  |
| 26873 | Cobutolin 2mg Tablet (Actavis UK Ltd) | Salbutamol sulfate | 1 |
| 27340 | Salbuvent 0.5mg/ml Injection (Pharmacia Ltd) | Salbutamol sulfate |  |
| 28881 | Salbutamol 2mg/5ml oral solution sugar free (A A H Pharmaceuticals Ltd) | Salbutamol sulfate |  |
| 29267 | Salbuvent 4mg Tablet (Pharmacia Ltd) | Salbutamol sulfate | 1 |
| 30118 | Salbutamol 100micrograms/dose inhaler CFC free (Teva UK Ltd) | Salbutamol sulfate |  |
| 30240 | Aerolin autohaler 100microgram/actuation Pressurised inhalation (3M Health Care Ltd) | Salbutamol sulfate |  |
| 31082 | Salbuvent 5mg/ml Respirator solution (Pharmacia Ltd) | Salbutamol sulfate |  |
| 31845 | Salapin 2mg/5ml syrup (Pinewood Healthcare) | Salbutamol sulfate |  |
| 32050 | Salbutamol 400 Cyclocaps (Teva UK Ltd) | Salbutamol sulfate |  |
| 32102 | Salbutamol 4mg tablets (A A H Pharmaceuticals Ltd) | Salbutamol sulfate | 1 |
| 33373 | Salbutamol 200 Cyclocaps (Teva UK Ltd) | Salbutamol sulfate |  |
| 33817 | Salbutamol 100micrograms/dose inhaler CFC free (Actavis UK Ltd) | Salbutamol sulfate |  |
| 34018 | Salbutamol 5mg/2.5ml Nebuliser liquid (Galen Ltd) | Salbutamol sulfate |  |
| 34162 | Salbutamol 2.5mg/2.5ml Nebuliser liquid (Galen Ltd) | Salbutamol sulfate |  |
| 34310 | Salbutamol 100micrograms/dose inhaler CFC free (A A H Pharmaceuticals Ltd) | Salbutamol sulfate |  |
| 34618 | Salbutamol 2mg tablets (Actavis UK Ltd) | Salbutamol sulfate | 1 |
| 34619 | Salbutamol 100microgram/inhalation Inhalation powder (Kent Pharmaceuticals Ltd) | Salbutamol sulfate |  |
| 38097 | Salbutamol cyclocaps 200microgram Inhalation powder (DuPont Pharmaceuticals Ltd) | Salbutamol sulfate |  |
| 38416 | Salbutamol cyclocaps 400microgram Inhalation powder (DuPont Pharmaceuticals Ltd) | Salbutamol sulfate |  |
| 40709 | Salbutamol 2.5mg/2.5ml nebuliser liquid unit dose vials (A A H Pharmaceuticals Ltd) | Salbutamol sulfate |  |
| 41549 | Salbutamol 2mg Tablet (C P Pharmaceuticals Ltd) | Salbutamol sulfate | 1 |
| 41691 | Salbutamol 2mg/5ml oral solution sugar free (Sandoz Ltd) | Salbutamol sulfate |  |
| 45863 | Salbutamol 5mg/2.5ml Nebuliser liquid (Generics (UK) Ltd) | Salbutamol sulfate |  |
| 556 | Combivent inhaler (Boehringer Ingelheim Ltd) | Salbutamol sulfate/Ipratropium bromide |  |
| 12909 | Salbutamol 100micrograms/dose / Ipratropium 20micrograms/dose inhaler | Salbutamol sulfate/Ipratropium bromide |  |
| 8267 | Sodium cromoglicate 1mg/dose / Salbutamol 100micrograms/dose inhaler | Salbutamol sulfate/Sodium cromoglicate |  |
| 10360 | Aerocrom inhaler (Castlemead Healthcare Ltd) | Salbutamol sulfate/Sodium cromoglicate |  |
| 1346 | Salbutamol 0.05mg/ml injection | Salbutamol Sulphate |  |
| 1882 | Ventodisks 200microgram/blister Disc (Allen & Hanburys Ltd) | Salbutamol Sulphate |  |
| 1950 | Ventodisks 400microgram/blister Disc (Allen & Hanburys Ltd) | Salbutamol Sulphate |  |
| 3163 | Salbutamol 200micrograms disc | Salbutamol Sulphate |  |
| 5753 | Salbutamol 400micrograms disc | Salbutamol Sulphate |  |
| 28577 | Ventolin 50microgram/ml Injection (Allen & Hanburys Ltd) | Salbutamol Sulphate |  |
| 30204 | Salbutamol 200micrograms inahalation capsules | Salbutamol Sulphate |  |
| 30212 | Salbutamol cyclohaler | Salbutamol Sulphate |  |
| 30230 | Salbutamol 100micrograms/actuation breath actuated inhaler | Salbutamol Sulphate |  |
| 34029 | Salbutamol 400micrograms inahalation capsules | Salbutamol Sulphate |  |
| 2152 | Ipratropium bromide with salbutamol 20mcg + 100mcg | Salbutamol Sulphate/Ipratropium Bromide |  |
| 11046 | Ipratropium bromide with salbutamol 500micrograms + 2.5mg/2.5ml | Salbutamol Sulphate/Ipratropium Bromide |  |
| 12822 | Salbutamol 2.5mg with ipratropium bromide 500micrograms/2.5ml unit dose nebuilser solution | Salbutamol Sulphate/Ipratropium Bromide |  |
| 1801 | Ventide inhaler (GlaxoSmithKline UK Ltd) | Salbutamol/Beclometasone dipropionate |  |
| 11307 | Salbutamol 100micrograms/dose / Beclometasone 50micrograms/dose inhaler | Salbutamol/Beclometasone dipropionate |  |
| 465 | Salmeterol 25micrograms/dose inhaler | Salmeterol xinafoate |  |
| 549 | Serevent 25micrograms/dose inhaler (GlaxoSmithKline UK Ltd) | Salmeterol xinafoate |  |
| 719 | Salmeterol 50micrograms/dose dry powder inhaler | Salmeterol xinafoate |  |
| 910 | Serevent diskhaler 50microgram Inhalation powder (Glaxo Wellcome UK Ltd) | Salmeterol Xinafoate |  |
| 2224 | Serevent 50micrograms/dose Accuhaler (GlaxoSmithKline UK Ltd) | Salmeterol xinafoate |  |
| 3297 | Salmeterol 50micrograms disc | Salmeterol Xinafoate |  |
| 7268 | Serevent 25micrograms/dose Evohaler (GlaxoSmithKline UK Ltd) | Salmeterol Xinafoate |  |
| 7270 | Salmeterol 25micrograms/dose inhaler CFC free | Salmeterol Xinafoate |  |
| 665 | Seretide 100 Accuhaler (GlaxoSmithKline UK Ltd) | Salmeterol xinafoate/Fluticasone propionate |  |
| 3666 | Seretide 500 Accuhaler (GlaxoSmithKline UK Ltd) | Salmeterol xinafoate/Fluticasone propionate |  |
| 5558 | Salmeterol 50micrograms with fluticasone 500micrograms CFC free inhaler | Salmeterol Xinafoate/Fluticasone Propionate |  |
| 5864 | Salmeterol 25micrograms with fluticasone 250micrograms CFC free inhaler | Salmeterol Xinafoate/Fluticasone Propionate |  |
| 5942 | Salmeterol 50micrograms with fluticasone 250micrograms CFC free inhaler | Salmeterol Xinafoate/Fluticasone Propionate |  |
| 6569 | Salmeterol 25micrograms with fluticasone 125micrograms CFC free inhaler | Salmeterol Xinafoate/Fluticasone Propionate |  |
| 6616 | Salmeterol 25micrograms with fluticasone 50micrograms CFC free inhaler | Salmeterol Xinafoate/Fluticasone Propionate |  |
| 6938 | Salmeterol 50micrograms with fluticasone 100micrograms dry powder inhaler | Salmeterol Xinafoate/Fluticasone Propionate |  |
| 11410 | Fluticasone propionate 500micrograms/dose / Salmeterol 50micrograms/dose dry powder inhaler | Salmeterol xinafoate/Fluticasone propionate |  |
| 13273 | Fluticasone propionate 100micrograms/dose / Salmeterol 50micrograms/dose dry powder inhaler | Salmeterol xinafoate/Fluticasone propionate |  |
| 314 | Intal 5mg/dose inhaler (Aventis Pharma) | Sodium cromoglicate |  |
| 964 | Sodium cromoglicate 5mg/dose inhaler | Sodium cromoglicate |  |
| 1422 | Cromogen 5mg/dose inhaler (Teva UK Ltd) | Sodium cromoglicate |  |
| 1629 | Intal 20mg/2ml nebuliser solution unit dose vials (Aventis Pharma) | Sodium cromoglicate |  |
| 1683 | Intal 20mg Spincaps (Sanofi) | Sodium cromoglicate |  |
| 1728 | Cromogen 5mg/dose Easi-Breathe inhaler (Teva UK Ltd) | Sodium cromoglicate |  |
| 2158 | Sodium cromoglicate 5mg/dose breath actuated inhaler | Sodium cromoglicate |  |
| 2911 | Sodium cromoglicate 20mg inhalation powder capsules | Sodium cromoglicate |  |
| 3585 | Steri-neb cromogen 10mg/ml Nebuliser liquid (IVAX Pharmaceuticals UK Ltd) | Sodium cromoglicate |  |
| 4100 | Intal autohaler 5 5mg/inhalation Pressurised inhalation (Aventis Pharma) | Sodium cromoglicate |  |
| 4647 | Intal 5mg/dose Syncroner with spacer (Aventis Pharma) | Sodium cromoglicate |  |
| 7972 | Intal 5mg/dose Fisonair with spacer (Sanofi) | Sodium cromoglicate |  |
| 8498 | Sodium cromoglicate 20mg/2ml nebuliser liquid unit dose vials | Sodium cromoglicate |  |
| 14603 | Sodium cromoglicate 5mg/dose inhaler with spacer | Sodium cromoglicate |  |
| 15765 | Sodium cromoglicate 5mg/inhalation inhaler & spacer | Sodium Cromoglicate |  |
| 37615 | Sodium cromoglicate 1mg/inhalation inhaler | Sodium Cromoglicate |  |
| 18314 | Aerocrom Syncroner with spacer (Castlemead Healthcare Ltd) | Sodium cromoglicate/Salbutamol sulfate |  |
| 24380 | Sodium cromoglicate 1mg/dose / Salbutamol 100micrograms/dose inhaler with spacer | Sodium cromoglicate/Salbutamol sulfate |  |
| 235 | Bricanyl 250micrograms/dose inhaler (AstraZeneca UK Ltd) | Terbutaline sulfate |  |
| 907 | Bricanyl turbohaler 500 500microgram Turbohaler (AstraZeneca UK Ltd) | Terbutaline sulfate |  |
| 1619 | Terbutaline 500micrograms/dose dry powder inhaler | Terbutaline sulfate |  |
| 1620 | Terbutaline 250micrograms/dose inhaler | Terbutaline sulfate |  |
| 4222 | Bricanyl 10mg/ml respirator solution (AstraZeneca UK Ltd) | Terbutaline sulfate |  |
| 4541 | Bricanyl SA 7.5mg tablets (AstraZeneca UK Ltd) | Terbutaline sulfate | 1 |
| 4640 | Bricanyl 5mg/2ml Nebuliser liquid (AstraZeneca UK Ltd) | Terbutaline sulfate |  |
| 5308 | Terbutaline 5mg/2ml nebuliser liquid unit dose vials | Terbutaline sulfate |  |
| 7711 | Terbutaline 250micrograms/dose inhaler with spacer | Terbutaline sulfate |  |
| 7954 | Bricanyl 250micrograms/dose spacer inhaler (AstraZeneca UK Ltd) | Terbutaline sulfate |  |
| 8522 | Terbutaline 7.5mg modified-release tablets | Terbutaline sulfate | 1 |
| 8676 | Terbutaline 10mg/ml nebuliser liquid | Terbutaline sulfate |  |
| 17874 | Monovent 1.5mg/5ml Oral solution (Lagap) | Terbutaline sulfate |  |
| 35522 | Bricanyl 500micrograms/1ml solution for injection ampoules (AstraZeneca UK Ltd) | Terbutaline sulfate |  |
| 35744 | Bricanyl 2.5mg/5ml solution for injection ampoules (AstraZeneca UK Ltd) | Terbutaline sulfate |  |
| 35862 | Terbutaline 500micrograms/1ml solution for injection ampoules | Terbutaline sulfate |  |
| 37612 | Terbutaline 5mg/2ml nebuliser liquid unit dose vials (Galen Ltd) | Terbutaline sulfate |  |
| 38419 | Terbutaline 1.5mg/5ml oral solution sugar free (A A H Pharmaceuticals Ltd) | Terbutaline sulfate |  |
| 41832 | Monovent 1.5mg/5ml syrup (Sandoz Ltd) | Terbutaline sulfate |  |
| 1628 | Terbutaline 250micrograms/actuation refill canister | Terbutaline Sulphate |  |
| 2758 | Bricanyl Refill canister (AstraZeneca UK Ltd) | Terbutaline Sulphate |  |
| 13307 | Bricanyl 500microgram/ml Injection (AstraZeneca UK Ltd) | Terbutaline Sulphate |  |
| 14483 | Terbutaline 500micrograms/ml injection | Terbutaline Sulphate |  |
| 15483 | Bricanyl Oral solution (AstraZeneca UK Ltd) | Terbutaline Sulphate/Guaifenesin |  |
| 17875 | Terbutaline with guafenesin expectorant | Terbutaline Sulphate/Guaifenesin |  |
| 26987 | Bricanyl Tablet (AstraZeneca UK Ltd) | Terbutaline Sulphate/Guaifenesin | 1 |
| 863 | Slo-phyllin 125mg Capsule (Lipha Pharmaceuticals Ltd) | Theophylline | 1 |
| 879 | Theophylline 125mg modified-release capsules | Theophylline | 1 |
| 880 | Theophylline 60mg modified-release capsules | Theophylline | 1 |
| 1097 | Slo-phyllin 60mg Capsule (Lipha Pharmaceuticals Ltd) | Theophylline | 1 |
| 1423 | Uniphyllin Continus 200mg tablets (Napp Pharmaceuticals Ltd) | Theophylline | 1 |
| 1832 | Theograd 350mg Tablet (Abbott Laboratories Ltd) | Theophylline | 1 |
| 1833 | Theophylline 200mg modified-release tablets | Theophylline | 1 |
| 1834 | Theophylline 400mg modified-release tablets | Theophylline | 1 |
| 2147 | Theophylline 250mg modified-release capsules | Theophylline | 1 |
| 2757 | Slo-phyllin 250mg Capsule (Lipha Pharmaceuticals Ltd) | Theophylline | 1 |
| 2995 | Nuelin SA 175mg tablets (Meda Pharmaceuticals Ltd) | Theophylline | 1 |
| 3388 | Theophylline 175mg modified-release tablets | Theophylline | 1 |
| 4593 | Theophylline 125mg tablets | Theophylline | 1 |
| 5261 | Nuelin SA 250 tablets (Meda Pharmaceuticals Ltd) | Theophylline | 1 |
| 5453 | Uniphyllin Continus 400mg tablets (Napp Pharmaceuticals Ltd) | Theophylline | 1 |
| 5941 | Uniphyllin Continus 300mg tablets (Napp Pharmaceuticals Ltd) | Theophylline | 1 |
| 6315 | Slo-Phyllin 250mg capsules (Merck Serono Ltd) | Theophylline | 1 |
| 7730 | Theo-Dur 300mg tablets (AstraZeneca UK Ltd) | Theophylline | 1 |
| 7731 | Theo-Dur 200mg tablets (AstraZeneca UK Ltd) | Theophylline | 1 |
| 7732 | Theophylline 300mg modified-release tablets | Theophylline | 1 |
| 7733 | Theophylline 250mg modified-release tablets | Theophylline | 1 |
| 7841 | Nuelin 125mg tablets (3M Health Care Ltd) | Theophylline | 1 |
| 9092 | Theophylline 350mg modified release tablets | Theophylline | 1 |
| 11719 | Slo-Phyllin 60mg capsules (Merck Serono Ltd) | Theophylline | 1 |
| 11993 | Pro-vent 300mg Capsule (Wellcome Medical Division) | Theophylline | 1 |
| 12240 | Theophylline 300mg modified release capsules | Theophylline | 1 |
| 15284 | Slo-Phyllin 125mg capsules (Merck Serono Ltd) | Theophylline | 1 |
| 21769 | Lasma 300mg Tablet (Pharmax Ltd) | Theophylline | 1 |
| 24418 | Biophylline 350mg Tablet (Lorex Synthelabo Ltd) | Theophylline | 1 |
| 24674 | Biophylline 500mg Tablet (Lorex Synthelabo Ltd) | Theophylline | 1 |
| 38120 | Theophylline 500mg modified release tablets | Theophylline | 1 |
| 10331 | Nuelin 60mg/5ml liquid (3M Health Care Ltd) | Theophylline sodium glycinate | 1 |
| 10433 | Theophylline 60mg/5ml oral solution | Theophylline sodium glycinate | 1 |
| 10723 | Theophylline 125mg/5ml syrup | Theophylline Sodium Glycinate | 1 |
| 10831 | Biophylline 125mg/5ml Oral solution (Lorex Synthelabo Ltd) | Theophylline Sodium Glycinate | 1 |
| 15365 | Theophylline 10mg/5ml SF elixir | Theophylline Sodium Glycinate | 1 |
| 27249 | Do-Do ChestEze tablets (Novartis Consumer Health UK Ltd) | Theophylline/Caffeine/Ephedrine hydrochloride | 1 |
| 2609 | Franol tablets (Sanofi) | Theophylline/Ephedrine hydrochloride | 1 |
| 12274 | Tedral Tablet (Parke-davis Research Laboratories) | Theophylline/Ephedrine Hydrochloride | 1 |
| 15153 | Theophylline 120mg / Ephedrine hydrochloride 11mg tablets | Theophylline/Ephedrine hydrochloride | 1 |
| 27944 | Tedral Oral solution (Parke-davis Research Laboratories) | Theophylline/Ephedrine Hydrochloride |  |
| 26860 | Theophylline 120mg / Ephedrine sulfate 15mg tablets | Theophylline/Ephedrine sulfate | 1 |
| 746 | Tiotropium 18 microgram Capsule | Tiotropium Bromide |  |
| 6050 | Spiriva 18 microgram Capsule (Boehringer Ingelheim Ltd) | Tiotropium Bromide |  |
| 34995 | Spiriva 18microgram inhalation powder capsules with HandiHaler (Boehringer Ingelheim Ltd) | Tiotropium bromide |  |
| 35000 | Spiriva 18microgram inhalation powder capsules (Boehringer Ingelheim Ltd) | Tiotropium bromide |  |
| 35011 | Tiotropium bromide 18microgram inhalation powder capsules | Tiotropium Bromide Monohydrate |  |
| 35014 | Tiotropium bromide 18microgram inhalation powder capsules with device | Tiotropium Bromide Monohydrate |  |
| 1973 | Accolate 20mg tablets (AstraZeneca UK Ltd) | Zafirlukast | 1 |
| 7132 | Zafirlukast 20mg tablets | Zafirlukast | 1 |
| 218 | AMINOPHYLLINE 100 MG CAP |  | 1 |
| 273 | THEOPHYLLINE 200 MG CAP |  | 1 |
| 2395 | SALBUTAMOL 2 MG/5ML SYR |  |  |
| 3763 | TERBUTALINE RESPULES INH |  |  |
| 3838 | SALBUTAMOL 400MCG/BECLOMETH.100MCG R/CAP INH |  | 1 |
| 4306 | BECLOMETHASONE DIPROPRIONATE CART 100 MCG |  |  |
| 7576 | BECLOMETHASONE DIPROPRIONATE CART 200 MCG |  |  |
| 8339 | FENOTEROL HYDROBROMIDE COMPLETE UNIT INH |  |  |
| 8470 | AMINOPHYLLINE 225 MG SUP |  |  |
| 8610 | AMINOPHYLLINE 1 GM SUP |  |  |
| 8653 | AMINOPHYLLINE 360 MG SUP |  |  |
| 8955 | THEOPHYLLINE 100 MG TAB |  | 1 |
| 10289 | AMINOPHYLLINE 200 MG SUP |  |  |
| 10432 | THEOPHYLLINE 300 MG SUP |  |  |
| 10744 | THEOPHYLLINE 80 MG ELI |  |  |
| 10958 | SALBUTAMOL .25 MG INJ |  |  |
| 15025 | AMINOPHYLLINE 25 MG SUP |  |  |
| 15409 | THEOPHYLLINE 3 MG SOL |  |  |
| 15441 | FENOTEROL HYDROBROMIDE .5 % SOL |  |  |
| 18308 | AMINOPHYLLINE 100 MG SUP |  |  |
| 19350 | AMINOPHYLLINE 62.5 MG SUP |  |  |
| 20171 | AMINOPHYLLINE 180 MG SUP |  |  |
| 20225 | AMINOPHYLLINE 500 MG INJ |  |  |
| 22080 | AMINOPHYLLINE 20 ML INJ |  |  |
| 24117 | AMINOPHYLLINE 300 MG SUP |  |  |
| 24207 | AMINOPHYLLINE PAED 50 MG SUP |  |  |
| 25022 | AMINOPHYLLINE 150 MG SUP |  |  |
| 25937 | AMINOPHYLLINE INTRAMUSCULAR 500 MG INJ |  |  |
| 27593 | AMINOPHYLLINE 350 MG SUP |  |  |
| 27842 | AMINOPHYLLINE 2 ML INJ |  |  |
| 29475 | BECLOMETHASONE DIPROPIONATE .02 ML LOT |  |  |
| 31231 | BECLOMETHASONE DIPROPRIONATE .5 % CRE |  |  |
| 32893 | THEOPHYLLINE 100MG/LYSINE 74MG MG TAB |  | 1 |

Appendix 4: Description of conditions and definitions of chronic conditions included in the multimorbidity score

| **CONDITION** | **DEFINITION** |
| --- | --- |
| Alcohol problems | Read code ever recorded |
| Anorexia or bulimia | Read code ever recorded |
| Anxiety & other neurotic, stress related & somatoform disorders | Read code in last 12 months OR ≥ 4 anxiolytic/hypnotic prescriptions in last 12 months |
| Asthma (currently treated) | Read code ever recorded AND any prescription in the last 12 months. |
| Atrial fibrillation | Read code ever recorded |
| Blindness and low vision | Read code ever recorded |
| Bronchiectasis | Read code ever recorded |
| Cancer - New diagnosis in last five years | Read code first recorded in last 5 years |
| Chronic kidney disease | Read Code ever recorded OR if the best (highest value) of the last 2 eGFR readings is less than 60 mL/min |
| Chronic Liver Disease and Viral Hepatitis | Read code ever recorded |
| Chronic sinusitis | Read code ever recorded |
| Constipation (Treated) | ≥4 laxative prescriptions in last year |
| COPD | Read code ever recorded |
| Coronary heart disease | Read code ever recorded |
| Dementia | Read code ever recorded |
| Depression | Read code recorded in last 12 months OR ≥4 anti-depressant prescriptions (excluding low dose tricyclics) in last 12 months |
| Diabetes | Read code ever recorded |
| Diverticular disease of intestine | Read code ever recorded |
| Epilepsy (currently treated) | Read code ever recorded AND antiepileptic prescription in last 12 months |
| Hearing loss | Read code ever recorded |
| Heart failure | Read code ever recorded |
| Hypertension | Read code ever recorded |
| Inflammatory bowel disease | Read code ever recorded |
| Irritable bowel syndrome | Read code ever recorded OR ≥4 antispasmodic prescription only in the last 12 months |
| Learning disability | Read code ever recorded |
| Migraine | ≥4 prescription only medicine anti-migraine prescriptions in last year |
| Multiple sclerosis | Read code ever recorded |
| Painful condition | ≥4 POM analgesics in last 12 months OR (≥4 specified anti-epileptics in last 12 months in the absence of an epilepsy Read code ever recorded) |
| Peripheral vascular disease | Read code ever recorded |
| Parkinson’s disease | Read code ever recorded |
| Prostate disorders | Read code ever recorded |
| Psoriasis or eczema | Read code ever recorded AND ≥4 related prescriptions in last 12 months (excluding |
| Psychoactive substance misuse (NOT ALCOHOL) | Read code ever recorded |
| Rheumatoid arthritis, other inflammatory polyarthropathies & systematic connective tissue disorders | Read code ever recorded |
| Schizophrenia (and related non-organic psychosis) or bipolar disorder | Read code ever recorded OR Lithium ever recorded |
| Stroke & transient ischaemic attack | Read code ever recorded |
| Thyroid disorders | Read code ever recorded |

Appendix 5: Episode and measures of practice variance in medication prescribed within three days of ALRTI^a^ diagnosis

|  |  | Ever diagnosed with asthma AND asthma medication prescribed 5 years prior to ALRTI | | | Ever diagnosed with asthma | | | |
| --- | --- | --- | --- | --- | --- | --- | --- | --- |
|  |  | Patients with asthma | Patients without asthma | | Patients with asthma | | Patients without asthma | |
| *ALRTI episodes, n=127,976 (n, %)* | | 27,719 (21.2) | 100,257 (78.3) | | 39,087 (30.5) | | 88,889 (69.5) | |
| No treatment | | 5,152 (18.6) | 18,531 (18.5) | | 9,389 (24.0) | | 14,305 (16.1) | |
| Antibiotics only^b^ | | 11,587 (41.8) | 68,469 (68.3) | | 16,469 (42.1) | | 63,679 (71.6) | |
| ^c^ Asthma medication only | | 702 (2.5) | 1,593 (1.6) | | 969 (2.5) | | 1,315 (1.5) | |
|  | Oral steroid^d^ | 380 (1.4) | 717 (0.7) | | 505 (1.3) | | 583 (0.7) | |
| Antibiotics^b^ and asthma medication^c^ | | 10,278 (37.1) | 116,64 (11.6) | | 12,260 (31.4) | | 9,590 (10.8) | |
|  | Antibiotics^b^ and oral steroids^d^ | 6,322 (22.8) | 5,279 (5.3) | | 7,290 (18.7) | | 4,248 (4.8) | |
| *Practice variance, n=513 (95% mid-range OR, 95% CI) ^f^* | | | |  | |  | |  |
| No treatment | | 2.04 (1.74, 2.51) | 2.05 (1.82, 2.38) | | 1.91 (1.68, 2.24) | | 2.02 (1.78, 2.36) | |
| Antibiotics only^b^ | | 1.51 (1.38, 1.69) | 1.96 (1.77, 2.21) | | 1.68 (1.52, 1.91) | | 2.05 (1.83, 2.34) | |
| Asthma medication only^c^ | | 6.57 (3.9, 13.56) | 23.16 (12.68, 48.79) | | 11.30 (5.26, 34.54) | | 31.00 (15.66, 72.68) | |
|  | Oral steroid^d^ | 19.01 (7.52, 73.54) | 155.52 (47.47, 733.78) | | 70.16 (15.13, 774.2) | | 180.80 (49.86, 1,002.39) | |
| Antibiotics^b^ and asthma medication^c^ | | 1.50 (1.37, 1.69) | 2.85 (2.42, 3.46) | | 1.37 (1.24, 1.57) | | 3.30 (2.73, 4.12) | |
|  | Antibiotics^b^ and oral steroids^d^ | 2.77 (2.28, 3.53) | 6.92 (5.06, 10.04) | | 2.86 (2.18, 4.12) | | 8.89 (6.21, 13.67) | |

IQR: interquartile range

^a^ Acute lower respiratory tract infection

^b^ Antibiotics limited to oral formulations and includes amoxicillin, doxycycline, clarithromycin, co-amoxiclav, azithromycin, erythromycin, tetracycline, cefalexin, cefradine.

^c^ Among patients without asthma, asthma medication refers to any asthma medication, including oral corticosteroids. Among patients with asthma, asthma medication refers to an increase in the dose of current treatment and/or additional asthma medication (including oral corticosteroids) prescribed compared to the prior month.

^d^ Oral steroids refer to oral corticosteroids

^e^ Practice variance was calculated from the variance of the random effect (σ2) and is given by e2×1.96×σ and represents the odds ratio comparing a practice at the 2.5th percentile of the distribution of practices to one at the 97.5th percentile for the treatment outcome of interest. For example, amongst patients without asthma, practices who most frequently prescribed no treatment were 2.14 times more likely to prescribe no treatment compared to practices who prescribed no treatment the least.

Appendix 6: Sensitivity analysis: multivariate final models investigating factors associated with an antibiotic prescription or change in asthma medication within three days of an ALRTI^a^ episode, stratified by asthma status^b^ and restricted to patients first ALRTI infection in the study period

|  |  | Patients with asthma (n=11,676) | | | | | | | Patients without asthma (n=46,767) | | | | |
| --- | --- | --- | --- | --- | --- | --- | --- | --- | --- | --- | --- | --- | --- |
|  |  | Antibiotic | | | Asthma med | | | Antibiotic | | | | Asthma med | |
|  |  | OR (95% CI) | p-value | OR (95% CI) | | p-value | OR (95% CI) | | | p-value | OR (95% CI) | | p-value |
| ***Demographic factors*** |  |  |  |  | |  |  | | |  |  | |  |
| Gender | Male | 1 (ref) |  | 1 (ref) | |  | 1 (ref) | | |  | 1 (ref) | |  |
|  | Female | 0.68 (0.56, 0.83) | <0.001 | 1.06 (0.97, 1.16) | | 0.21 | 0.77 (0.71, 0.84) | | | <0.001 | 1.12 (1.03, 1.20) | | 0.001 |
| Age at ALRTI (years) | Per SD | 1.05 (0.95, 1.16) | 0.36 |  | |  | 1.00 (0.96, 1.05) | | | 0.99 |  | |  |
|  | Q1 (<39) |  |  | 1 (ref) | |  |  | | |  | 1 (ref) | |  |
|  | Q2 (39-) |  |  | 0.96 (0.85, 1.09) | |  |  | | |  | 1.08 (0.97, 1.19) | |  |
|  | Q3 (52-) |  |  | 0.87 (0.77, 0.99) | |  |  | | |  | 0.84 (0.76, 0.93) | |  |
|  | Q4 (64+) |  |  | 0.76 (0.67, 0.87) | | <0.001 |  | | |  | 0.61 (0.54, 0.68) | | <0.001 |
| IMD quintile | 1 (Least) | 1 (ref) |  | 1 (ref) | |  | 1 (ref) | | |  | 1 (ref) | |  |
|  | 2 | 1.35 (1.02, 1.79) |  | 0.90 (0.78, 1.04) | |  | 0.96 (0.84, 1.09) | | |  | 0.92 (0.82, 1.04) | |  |
|  | 3 | 1.03 (0.98, 1.73) |  | 1.08 (0.94, 1.24) | |  | 0.90 (0.79, 1.03) | | |  | 1.04 (0.92, 1.18) | |  |
|  | 4 | 1.21 (0.91, 1.60) |  | 0.98 (0.84, 1.13) | |  | 0.88 (0.76, 1.01) | | |  | 0.93 (0.81, 1.05) | |  |
|  | 5 (Most) | 1.57 (1.16, 2.13) | 0.05 | 0.94 (0.81, 1.10) | | 0.12 | 0.99 (0.86, 1.15) | | | 0.22 | 0.97 (0.85, 1.11) | | 0.20 |
| Current smoking status | Never | 1 (ref) |  | 1 (ref) | |  | 1 (ref) | | |  | 1 (ref) | |  |
|  | Current | 1.01 (0.83, 1.24) |  | 1.14 (1.03, 1.26) | |  | 1.05 (0.96, 1.15) | | |  | 1.12 (1.03, 1.22) | |  |
|  | Previous | 0.86 (0.67, 1.10) | 0.40 | 1.04 (0.92, 1.18) | | 0.03 | 1.20 (1.05, 1.37) | | | 0.03 | 1.01 (0.90, 1.14) | | 0.01 |
| ***Clinical factors*** |  |  |  |  | |  |  | | |  |  | |  |
| Prescribed 'step-up' in asthma medication ^c^ | No | 1 (ref) |  |  | |  | 1 (ref) | | |  |  | |  |
|  | Yes | 1.41 (1.28, 1.55) | <0.001 | ^h^ | |  | 0.93 (0.83, 1.03) | | | 0.17 | ^h^ | |  |
| Prescribed an antibiotic ^d^ | No |  |  | 1 (ref) | |  |  | | |  | 1 (ref) | |  |
|  | Yes | ^h^ |  | 3.69 (3.17, 4.29) | | <0.001 | ^h^ | | |  | 2.30 (2.04, 2.58) | | <0.001 |
|  |  |  |  |  | |  |  | | |  |  | |  |
| Number of ALRTI in prior 12 months, per ALRTI | Per SD |  |  |  | |  |  | | |  | 0.94 (0.78, 1.12) | | 0.48 |
|  | 0 | 1 (ref) |  | 1 (ref) | |  | 1 (ref) | | |  |  | |  |
|  | 1 | 0.86 (0.52, 1.43) |  | 1.05 (0.82, 1.34) | |  | 0.38 (0.3,0 0.47) | | |  |  | |  |
|  | 2+ | 0.42 (0.16, 1.11) | 0.19 | 1.23 (0.68, 2.20) | | 0.741 | 0.51 (0.28, 0.91) | | | <0.001 |  | |  |
| Multimorbidity score ^e^ | 0 | 1 (ref) |  | 1 (ref) | |  | 1 (ref) | | |  | 1 (ref) | |  |
|  | 1 | 1.07 (0.84, 1.37) |  | 1.02 (0.91, 1.14) | |  | 1.03 (0.92, 1.14) | | |  | 1.09 (0.99, 1.19) | |  |
|  | 2 | 1.24 (0.93, 1.66) |  | 0.98 (0.86, 1.12) | |  | 0.99 (0.87, 1.12) | | |  | 1.00 (0.89, 1.12) | |  |
|  | 3+ | 0.94 (0.72, 1.21) | 0.24 | 0.81 (0.71, 0.92) | | 0.001 | 0.83 (0.73, 0.93) | | | 0.001 | 0.99 (0.89, 1.10) | | 0.24 |
| Number of antibiotics prescribed in previous 12 months ^f^ | 0 | 0.002 (0.001, 0.003) |  |  | |  | 0.003 (0.003, 0.003) | | |  |  | |  |
|  | 1 | 1 (ref) |  |  | |  | 1 (ref) | | |  |  | |  |
|  | 2 | 1.15 (0.91, 1.47) |  |  | |  | 0.97 (0.87, 1.08) | | |  |  | |  |
|  | 3+ | 0.99 (0.79, 1.22) | <0.001 | ^h^ | |  | 0.88 (0.79, 0.98) | | | <0.001 | ^h^ | |  |
| Number of asthma medications prescribed in previous 12 months ^c^ | 0 |  |  |  | |  |  | | |  | 1 (ref) | |  |
|  | 1-6 |  |  |  | |  |  | | |  | 1.63 (1.47, 1.80) | |  |
|  | 7+ |  |  |  | |  |  | | |  | 2.42 (1.89, 3.09) | | <0.001 |
|  | Q1 (1) |  |  | 1 (ref) | |  |  | | |  |  | |  |
|  | Q2 (2-) |  |  | 1.12 (0.98, 1.28) | |  |  | | |  |  | |  |
|  | Q3 (5-) |  |  | 1.00 (0.87, 1.15) | |  |  | | |  |  | |  |
|  | Q4 (10+) | ^h^ |  | 0.95 (0.82, 1.10) | | 0.029 | ^h^ | | |  |  | |  |
| ***Practice factors*** |  |  |  |  | |  |  | | |  |  | |  |
| Practice ALRTI antibiotic rates, per 1,000 patients ^g^ | per SD | 1.50 (1.37, 1.64) | <0.001 | ^h^ | |  | 1.49 (1.42, 1.57) | | | <0.001 | ^h^ | |  |

OR: odds ratio; CI: confidence interval; ref: reference

Models restricted to participants with complete information on all variables included in the full model. Multi-level logistic regression models were used, with general practice included as a random effect to account for clustering. Coefficients represent the odds of receiving an antibiotic/asthma medication for a unit increase in the exposure of interest.

^a^ Acute lower respiratory tract infection

^b^ Defined as ever diagnosed and asthma medication prescribed in the 12 months prior to ALRTI.

^c^ Asthma medication refers to an increase in the dose of current treatment and/or additional asthma medication prescribed compared to the prior month for patients with asthma, and any asthma medication for those without. Asthma medication includes long- and short-acting β2 agonists, leukotriene receptor antagonists, antimuscarinic bronchodilators, and inhaled and oral corticosteroids

^d^ Antibiotics prescribed within three days of ALRTI, and includes amoxicillin, doxycycline, clarithromycin, co-amoxiclav, azithromycin, erythromycin, tetracycline, cefalexin, and cefradine.

^e^ Any antibiotics (BNF chapter 5.1) prescribed in the last 12 months for any condition.

^f^ A list of 37 physical and mental chronic conditions were used to ascertain multimorbidity status in participants at ALRTI diagnosis.

^g^ Practice ALRTI antibiotic rates standardised (using sample mean values and SDs) and coefficients represent a change in the OR in a antibiotic prescribing/step-up in asthma medication per 1-standard deviation increment in exposure of interest.

^h^ Variable not relevant to model
